# North Sea demersal fisheries prefer specific benthic habitats

**DOI:** 10.1371/journal.pone.0208338

**Published:** 2018-12-18

**Authors:** Karin J. van der Reijden, Niels T. Hintzen, Laura L. Govers, Adriaan D. Rijnsdorp, Han Olff

**Affiliations:** 1 Groningen Institute for Evolutionary Life Sciences (GELIFES), University of Groningen, Groningen, the Netherlands; 2 Wageningen Marine Research, Ijmuiden, the Netherlands; 3 Department of Coastal Systems. Royal Netherlands Institute for Sea Research (NIOZ), Texel, the Netherlands; 4 Aquaculture and Fisheries, Wageningen University, Wageningen, the Netherlands; MARE – Marine and Environmental Sciences Centre, PORTUGAL

## Abstract

**Introduction:**

The future protection of marine biodiversity through good conservation planning requires both the identification of key habitats with unique ecological characteristics and detailed knowledge of their human utilization through fisheries. Demersal fisheries are important disturbers of benthic habitats. They often have a heterogeneous spatial distribution, pressurizing particular habitats with high abundances of target species. For the North Sea, we quantified the commonness/rarity of habitats in relation to the environmental determinants of so-called fishing hotspots, to support better-informed conservation planning of benthic habitats in this intensively used continental shelf.

**Methods:**

We first distinguished 9 main seascapes in the study area based on seabed morphology. Secondly, we determined average fishing intensity and fishing hotspots using VMS-data for the three dominant Dutch fisheries from 2008 to 2015: beam-trawlers targeting sole *Solea solea* (Beam-Sole), beam-trawlers targeting plaice *Pleuronectes platessa* (Beam-Plaice), and otter-trawlers targeting Norway lobster *Nephrops norvegicus* and demersal fish (Otter-Mix). Within the seascapes subjected to >80% of the fishing activity, nineteen environmental factors (summarized by PCA) were used to ecologically characterize fishing hotspot locations using MaxEnt response modelling.

**Results:**

We found that all three fisheries target highly specific, uncommon habitats. Beam-Sole fishers targeted warmer, shallow, dynamic, nearshore habitats, and within these specifically the depressions between sand ridges. Beam-Plaice fishers mainly targeted the exposed, non-muddy flanks of the Dogger Bank and similar large-scale elevations (50–75 km) where especially the ridges of smaller sand banks are used. Otter-Mix fisheries concentrated in areas with low bed shear stress, located in muddy, relatively deeper areas.

**Implications:**

This study is the first to provide insight in benthic habitat types that are frequently targeted by fishers in the North Sea. We demonstrated unequal exploitation pressure between seabed habitats, with the majority of hotspots in the less common habitats. Our results hence contribute to a more effective, ecologically informed planning for the protection and monitoring of all seabed habitats and biodiversity of the North Sea.

## Introduction

Marine conservation planning generally aims at protecting key and unique habitats and biodiversity, characterized by specific combinations of environmental conditions. To establish successful conservation measures such as novel marine protected areas, it is important to know both where these sites of special conservation interest are located, and how such locations are presently affected by anthropogenic impacts [1]. Benthic habitats can be subject to multiple human impacts, with demersal fisheries as one of the dominant stressors with long-term impacts on community structure and biodiversity [2,3]. On continental shelves, bottom trawling affects habitats not only through removal of the target species, but also by both causing mortality of unintended bycatch species and through their physical impact on the seafloor [4–7]. The contact of gear components as trawl doors and tickler chains with the seafloor causes direct damage to benthic organisms[8], resuspension of sediments [9,10] and the destruction of bioengineered epi- and endobenthic structures such as reefs and burrows [11]. The physical impact varies between different types of sediment [12], while the impact on benthic communities depends on the resilience of the community itself as well [4,13]. Good understanding of both the spatial distribution and the environmental drivers of demersal fisheries and benthic habitats is therefore necessary to find adequate management measures that minimize fisheries impacts on marine benthic communities and habitats. This helps answering the important question which habitats are now strongly impacted by human activities throughout their entire geographic range and which habitats also persist under conditions of low human impact.

It is known that demersal fishers target quite specific fishing grounds instead of homogeneously distributing their fishing effort [3,14–16]. Previous studies have linked these fishing grounds to fish aggregation patterns [17–20], primary production [21], seabed morphology [3], unsuitability of particular fishing gears for specific substrates [3], and legislator restrictions, as marine protected areas [22]. Nevertheless, a complete overview of physical and ecological characteristics of North Sea fishing grounds is lacking. Therefore, we do not know which combinations of environmental conditions (habitats) are currently most impacted by different types of fisheries, and which habitats are less affected. Since 2002, more detailed analyses of fisheries distributions are becoming possible, as all fishing vessels above a threshold (>24m since 2002, >15m since 2006, and >12 since 2012) are required to participate in the European Vessel Monitor System (VMS) [23], in addition to the obliged daily logbook registrations [24,25]. For the North Sea, a recent analysis of international VMS-data revealed that, over the three-year study period, trawling occurred in 93% of the 1 x 1 minute longitude and latitude grid cells. However, 90% of the fishing activity was concentrated in 45.4% of the area [3], showing the highly heterogeneous distribution of fishing activities. Within the set of grid cells of concentrated fishing effort, locations with yearly recurring high fishing activity can be observed, the so-called ‘hotspots’. The exact locations of these hotspots, however, are yet unknown.

Moreover, the key environmental factors that are associated with the stable fishing hotspots are not well understood [3], requiring an analysis at an even higher spatial resolution and with the inclusion of a large set of environmental predictors. We hypothesize that these stable hotspots are structured by small-scale variations in environmental conditions. This habitat heterogeneity could be created by oceanographic structures, such as depressions or elevations relative to the surrounding seabed, but also by the presence of smaller patches of specific sediment types, or particular hydrodynamic conditions. These conditions could then directly [26] or indirectly [27–29] result in stable aggregations of target species, attracting specific fisheries. Most likely, these stable species aggregations underlying the stable fishing hotspot locations are fisheries-specific, as each fishery is targeting different species. We therefore hypothesize that each fishery targets specific environmental conditions, resulting in unique, fisheries-specific sets of environmental conditions at the various stable hotspots.

In this study, we performed a detailed analysis of the environmental characteristics of the demersal fisheries hotspots in the Southern and Central North Sea by comparing prevailing conditions within and between fishing hotspots, but also with environmental conditions at non-hotspot locations. We identified seascapes, based on seabed morphology, and selected those subject to >80% of the fishing activity as main fishing ground. Within these fishing grounds, we determined the relevant multifactor environmental gradients with Principal Component Analysis (PCA), using 19 environmental variables. Maximum Entropy (MaxEnt) species distribution models were then applied to both stable fishing hotspot locations, based on VMS data for all Dutch vessels in the area from 2008 to 2015, and the environmental gradients [30]. Additionally, we quantified if the targeted environmental ranges reflect rare or common benthic habitats. We discuss how the yielded information can improve current management of marine resources.

## Materials and methods

### Study area and seascape determination

**Fig 1:**
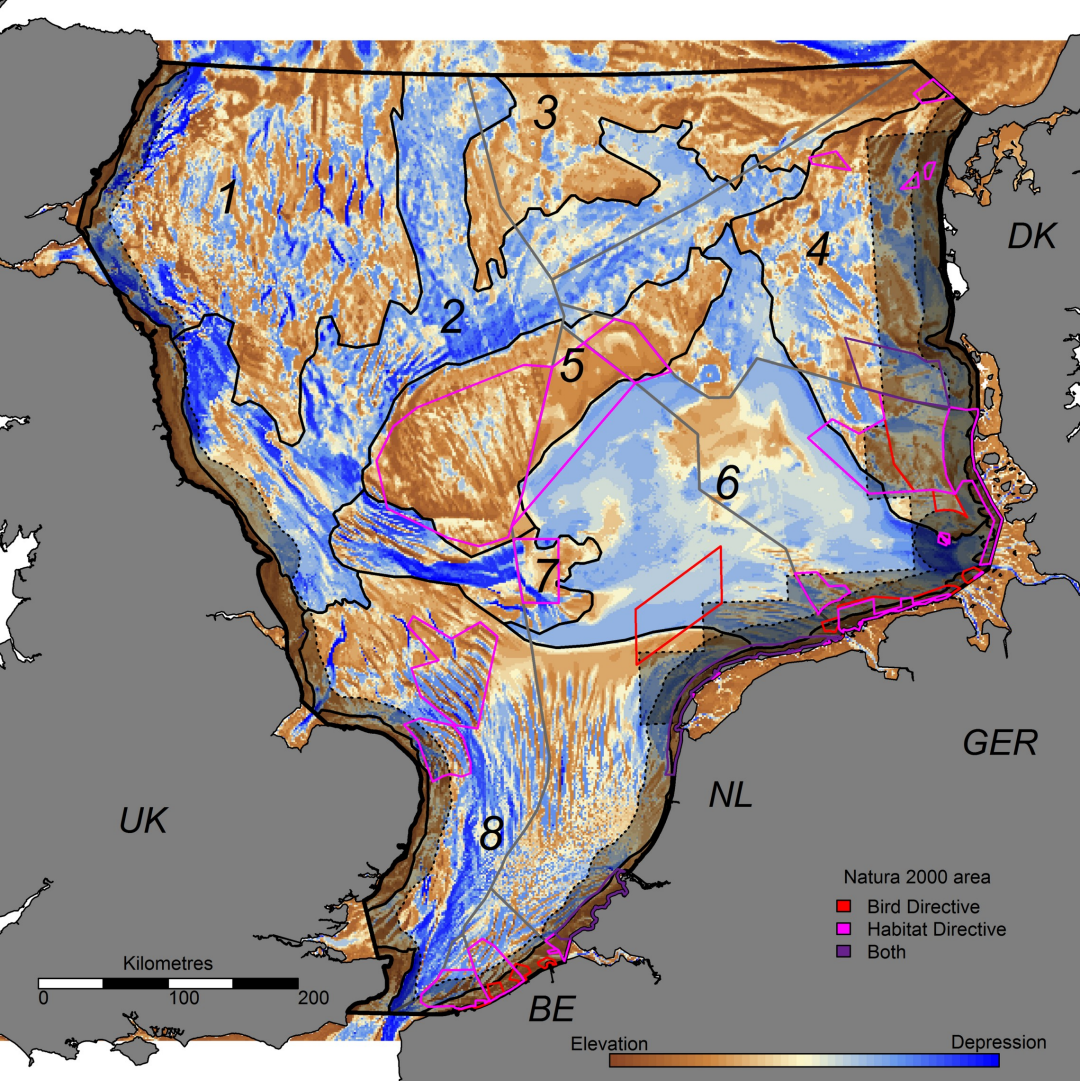
The study area with 9 seascapes. The seascapes comprise 8 (numbered) offshore seascapes and multiple coastal seascapes (not numbered). Brown values show elevated areas, while blue values show local depressions. The Plaice Box and the National Territorial Waters (12 nautical miles from the shore line) are shown as grey shaded areas with dotted contour, while the Exclusive Economic Zones are depicted with grey lines. Current Natura 2000 areas are displayed, with red lines showing protected areas under de Bird Directive, pink lines showing protected areas under the Habitat Directive. Purple lines outline protected areas under both the Bird and Habitat Directive.

We focus our study on the demersal fisheries by the Dutch fleet in the Southern and Central North Sea, defined as the combination of subdivisions IVb and IVc according to the International Council for the Exploration of the Sea (ICES; Fig 1). We subdivided this region in areas with similar seabed morphological characteristics, which we call seascapes. We determined seabed morphology using Bathymetry Positioning Indices (BPI), which depict the depth of a location (pixel) relative to the depth of its surrounding. This allows for the identification of underwater sand ridges, troughs, and relative flat areas. Using bathymetry data from the European Marine Observation and Data Network (EMODnet; www.emodnet.eu/bathymetry) with a resolution of ±178m, we calculated the relative depth of every pixel as the difference between the average depth of all pixels within a certain radius and its absolute depth [31]. The relative depth was classified into 5 classes; strong depression (< -10m), weak depression (-10m to -1m), flat (-1m to +1m), weak elevation (+1m to +10m), strong elevation (> +10m). Main seabed morphology was then determined as the weighted summation of the classified BPI with radii of 5, 10, 30, 50, and 75 km, giving reduced weight to BPI’s with smaller radii as shown in formula 1. This methodology was chosen as it emphasizes the large-scale seabed morphology, which is expected to have the largest impact on benthic communities [32]. The aforementioned seascapes were manually delineated in ArcGIS based on the main seabed morphology.

$$BPI_{75km}+0.9*BPI_{50km}+0.8*BPI_{30km}+0.7*BPI_{10km}+0.6*BPI_{5km}$$ (1)

### Main fishing grounds and fisheries hotspots

VMS and logbook data, covering all Dutch demersal fishing vessels >15m, were available from 2008 to 2015. VMS-data recordings comprise a satellite-based registration of the location, speeding and heading of the vessel, and the date and time, which are recorded with an interval of ± 2 hours. Logbook data additionally include information on the vessel and the gear used. These datasets are coupled for each unique combination of vessel and day within the fishing trip. Based on gear type, engine power, and mesh size, distinct fisheries were defined, which typically have a specific (set of) target species. Then, based on speed and gear, VMS pings are categorized as either ‘fishing’, ‘steaming’ or ‘floating [24,33]. To correct for heaving and shooting during fishing activity, ‘floating’ pings in between ‘fishing’ pings are reclassified to ‘fishing’ [34]. We calculated the swept area for each ping, as the product of speed (km/h), fishing time (h), and the width of the gear (km) [35]. At a grid of 1 km^2^, total swept surface was aggregated to calculate annual fishing intensity for each fishing gear. Taking the mean of the fishing intensities of each grid cell over the study period resulted in the average fishing distribution (average of the yearly swept area ratio (km^2^ year^-1^)). In this study, we restrict ourselves to the three types of fisheries that are internationally dominated by Dutch fishers and who have their main fishing grounds offshore. (1) Beam-Sole: beam-trawlers deploying nets with mesh sizes of 70-99mm, targeting sole *Solea solea* [36]. (2) Beam-Plaice: beam-trawlers with mesh sizes of 100mm or larger, mainly targeting plaice *Pleuronectes platessa* [37]. And (3) Otter-Mix: otter-trawlers with mesh sizes of 70-99mm, fishing for both demersal flatfish and Norway lobster [38]. To enable a complete overview of the Dutch demersal fishing activity distribution, however, we calculated the average fishing activity for the remaining demersal gears together as well.

For each seascape, the average fishing effort was calculated based on this average fishing distribution (Table 1). Fisheries-specific main fishing grounds were than determined as those seascapes in which the majority (>80%) of the fishing effort took place. Stable fishing hotspots, on the other hand, were defined as those individual grid cells (1x1 km) that belonged to the annual top 1% most fished grid cells in at least 7 out of the 8 year study period. Therefore, hotspots depict the stable areas with the highest fishing intensities (in yearly swept area (km^2^year^-1^). These hotspots corresponded to 12.1–13.8% (Beam-Sole), 1.7–4.8% (Beam-Plaice), and 29.9–56.5% (Otter-Mix) of the total annual fishing activity per fishery.

**Table 1:** Fishing activity per seascape for the Beam-Sole, Beam-Plaice, and Otter-Mix fishing categories.

	Seascape	1	2	3	4	5	6	7	8	9
	Surface (km^2^)	47341	52420	26114	36584	25214	55420	10266	58253	15259
**Beam-Sole**	Fishing effort (kW)	0	273	1	1758	463	**18135**	3058	**44763**	78
	Fishing effort (%)	*-*	*0*.*4*	*0*.*0*	*2*.*6*	*0*.*7*	***26*.*5***	*4*.*5*	***65*.*3***	*0*.*1*
	Hotspots present	0	0	0	0	0	**7**	7	**2632**	6
**Beam-Plaice**	Fishing effort (kW)	0	30	99	**1067**	**3159**	353	23	240	26
	Fishing effort (%)	*-*	*0*.*6*	*2*.*0*	***21*.*4***	***63*.*2***	*7*.*1*	*0*.*5*	*4*.*8*	*0*.*5*
	Hotspots present	0	0	0	**68**	**91**	1	0	0	0
**Otter-Mix**	Fishing effort (kW)	2	585	3	572	75	**15329**	**6817**	1035	356
	Fishing effort (%)	*0*.*0*	*2*.*4*	*0*.*0*	*2*.*3*	*0*.*3*	***61*.*9***	***27*.*5***	*4*.*2*	*1*.*4*
	Hotspots present	0	0	0	12	0	**2062**	**786**	6	1

Squares displayed in bold indicate seascapes that define the fisheries-specific study sites used in the MaxEnt models.

### Environmental data

**Fig 2:**
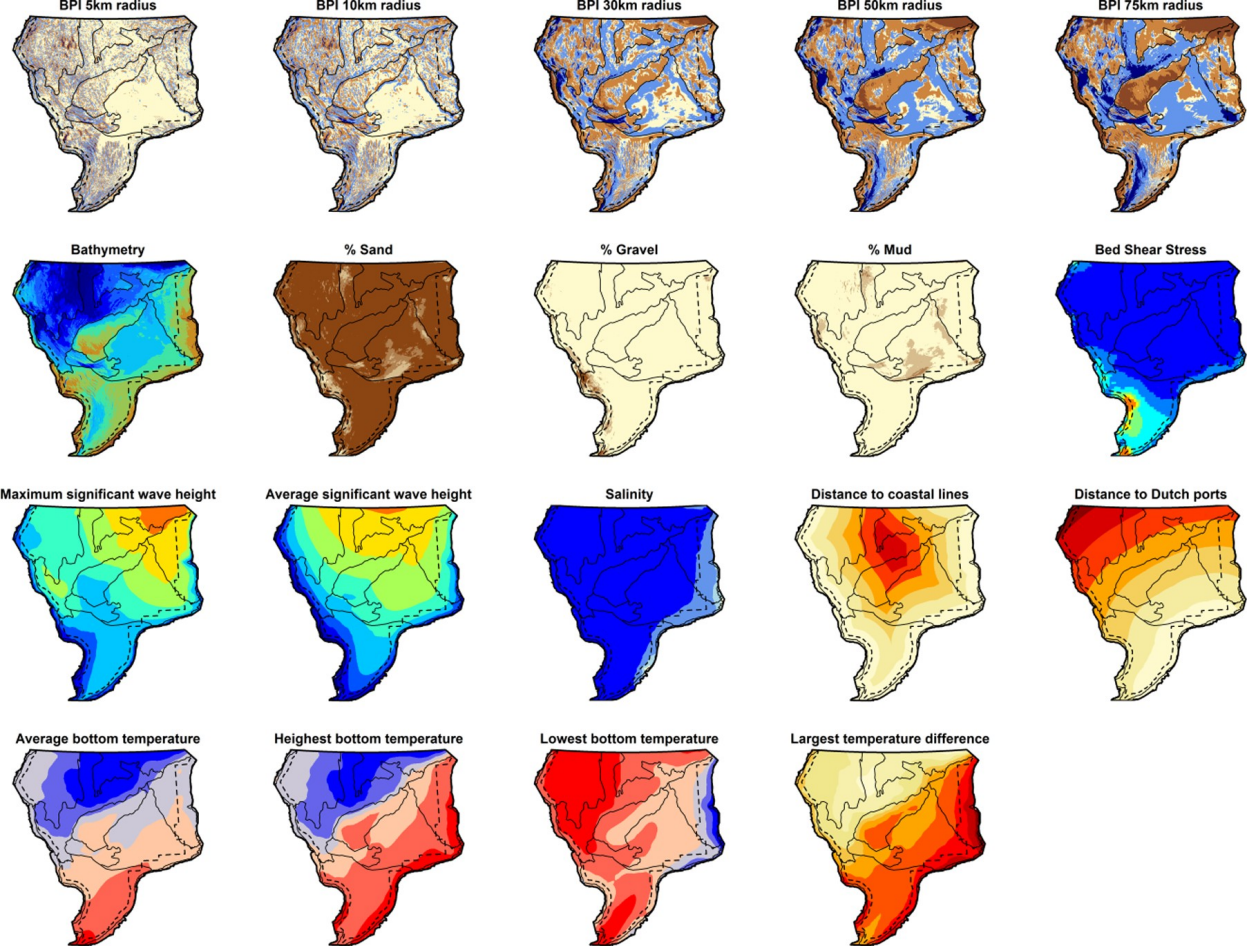
Overview of all environmental factors taken into consideration in this study. For the Bathymetric Position Indices (BPIs), a brown (relative elevation) to blue gradient (relative depression) is used to show seabed morphology. Bathymetry has a gradient of blue (deep waters) to green to brown (shallow). Higher sediment contents are depicted with darker brown colours, while Bed Shear Stress and wave action are shown from blue (low stress/action) to red (high stress/action). Higher salinity values show darker blue. Distance to nearest coast and port, as well as largest temperature difference range from small distance/difference (white) to large distance/difference (red). The bottom temperatures are depicted with blue representing the coolest temperatures towards the highest temperatures in red. All figures are individually included in the Supplementary Materials, including the range and associated legend.

Nineteen environmental variables were used, from different data sources (Fig 2). Bathymetry data for the entire North Sea is available from the EMODnet project (http://portal.emodnet-bathymetry.eu/) at a resolution of ±178x178m (S1 Fig). These data formed the basis for the BPI calculations (S2–S6 Figs). GeoTIFFs for gravel, mud and sand content of the sediment were obtained from Stephens (2015; S7–S9 Fig). Tidal bed shear stress (BSS; the force exerted on the seabed by tidal currents in N/m^2^) was obtained from a hydrodynamic model by John Aldridge (CEFAS) as used in [4,39] (S10 Fig). The daily significant wave height for 2015 was obtained from the North-West European Shelf Wave Analysis and Forecast system by MetOffice, at the European website Copernicus (http://marine.copernicus.eu/). Herewith, the maximum significant wave height and the average wave height were determined (S11 and S12 Figs). The same Copernicus website was used to simultaneously obtain monthly average modelled bottom salinity and temperature estimates of the seawater for 2008–2013 from the ocean physics reanalysis by MetOffice (S13 Fig). We used temperature data to determine average temperature (mean of all monthly temperatures), and the average annual minimum and maximum temperature (by first determining the minimum and maximum temperature within a year and averaging these over the study period). Additionally, the difference between average minimum and maximum temperature was calculated, as a measure for temperature variation (S14–S17 Figs). Finally, the shortest distance to a surrounding coastal line (S18 Fig) and a Dutch harbour with auction (S19 Fig) were calculated for all locations within the study area. The environmental variables were all obtained as raster-files, with varying resolution, extent and projection. Re-projection to the same ETRS89/UTM31 projection (EPSG: 25831) and a bilinear interpolation method were then applied to create a consistent set of environmental factor raster files, with similar characteristics as the abiotic factor with the highest resolution (depth; ±178x178m).

### Data analysis

#### Ordination of environmental factors

Fishermen have practical considerations in choosing their fishing ground, with a trade-off between travelling (fuel) costs and expected yield [33,40]. To ensure that we only include relevant ranges of environmental factors in the analysis, we restricted our analysis to the conditions prevailing at the main fishing grounds (seascapes with >80% of the fishing activity; Table 1) [41]. This also enables the potential identification of preferences for minor deviating habitats, which would be overshadowed by the distance-to-shore gradient otherwise. For each fishing ground, we extracted the environmental gradient values at the midpoints of all enclosed grid cells. Any potential prejudice in importance (weight) of specific environmental factors was excluded by the separate inclusion of all environmental factors. The values were than scaled and centered, and a Principal Component Analysis (PCA) was performed. This reduced the number of dimensions, and yielded an ecological relevant interpretation of the different sets of correlated environmental factors important within that specific fishing ground. Components with eigenvalues >1 were kept [42], representing sets of correlated factors here-after referred to as environmental gradients. For each environmental gradient, a frequency distribution of grid cell values was calculated to determine range distribution over the main fishing ground. This can be interpreted as the rarity of a specific gradient value within its range for the area.

#### Spatial autocorrelation

The likelihood that two points are similar depends on the distance between them, with more similar points located closer together. To ensure that our analysis was not affected by such spatial auto-correlation, we tested for spatial auto-correlation by applying a variogram (package “gstat”) to the midpoints of the grid cells [43]. A variogram calculates the correlation between the points and plots these, sorted for the distance between the points. Herewith, the minimum distance between two points to have no auto-correlation (range) can be determined. We set the upper limits of our variograms to a distance of <20 km, as others [15] found less than 5% similarity between fishing locations with this distance. The best fitted variogram-model was selected, and its range determined. This range was then used as minimum distance in a resampling of the hotspot locations.

#### Species distribution models

The relation of fishing hotspots with environmental gradients was determined using a MaxEnt species distribution model [29,43], one of the various ecological niche modelling methods currently available for presence-only data (MaxEnt version 3.3.3k). Combining species presences and background locations with a set of (uncorrelated) environmental predictors, a relative occurrence rate (ROR) is calculated. By applying a logistic output to this ROR and by assuming random spatial sampling for species presence, species presence probability can be estimated [40]. Moreover, the model calculates the correlation between environmental predictors and the species distribution, allowing for complex, nonlinear responses. We specifically applied a MaxEnt model for this ability, because these curves provide a realistic correlation between fishing hotspot location and their environmental conditions, although they can be hard to interpret [40].

Here, we compared conditions at fishing hotspots (presences) with the overall environment, by randomly creating background locations among all non-hotspot grid-cells within the main fishing ground. These background locations had a similar minimum distance and exceeded the number of presence locations by a factor 5 [44]. Environmental gradient values were extracted for the midpoints of the grid cells defined as hotspot or background and supplied to the MaxEnt model. With these, the model constructed the most optimal response curves in a single run, allowing for linear, quadratic, threshold and hinge features [40]. Product features, which are comparable to interactive functions between separate environmental gradients, were set to not-allowed.

## Results

### Seascapes

Based on the similarity of seabed morphology features (as determined by the weighted summation of five bathymetry position indices (Eq 1)), we determined nine seascapes in the study area (Fig 1). Seascapes 1 and 3 show elevated areas, whereas seascape 2 is relatively lower compared to its surroundings. Seascape 1 is characterized by strong relief at the 5–10 km scale; seascape 3 has little of such relief. Seascape 4 is separated in the north from seascape 3 and from seascape 6 (Central Oyster Grounds) in the west by a depression. Seascape 5, which comprises the Dogger Bank area, is strongly elevated compared to its surroundings, whereas seascape 7 (the Cleaver Bank) is characterized by some very low areas. Seascape 8 shows high, well-structured relief at the small-scale (5-10km), which is caused by tidal ridges. Seascape 9 is the only seascape comprising multiple separate areas, but all located near the coast and characterized by locally elevated areas.

### Main fishing grounds and fisheries hotspots

**Fig 3:**
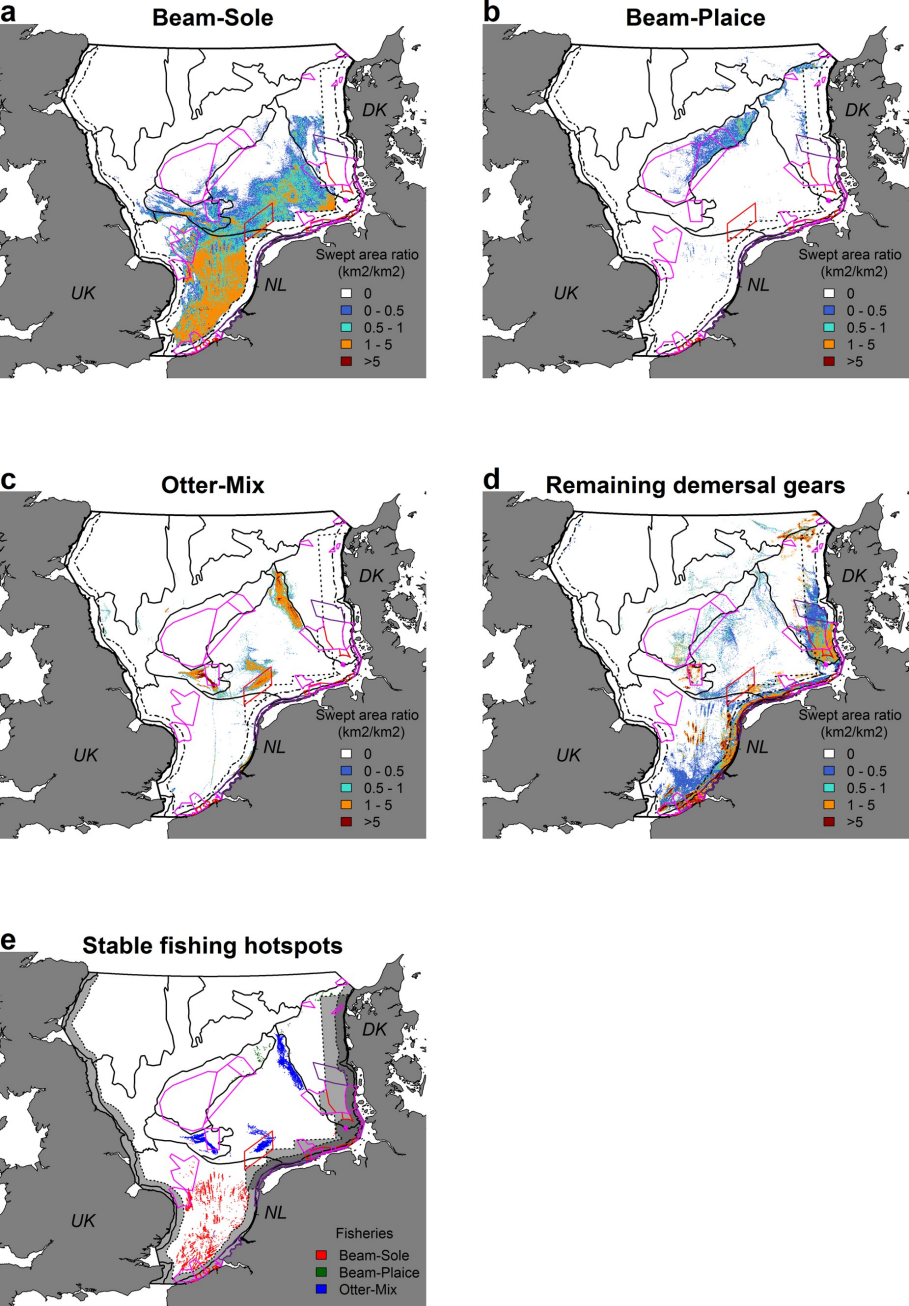
**Average fishing intensity of A) Beam-Sole, B) Beam-Plaice, C) Otter-Mix, and D) the remaining demersal gears in the Dutch fleet over the time period 2008–2015.** For the three gear types of interest, the stable fishing hotspots are depicted in E. Fishing intensity is calculated as the averaged fished area in km^2^ per year. Stable fishing hotspots are those grid cells of 1km^2^ that belong to the annual 1% most intense used grid cells at least 7 times over the study period (no unit). Black solid lines represent the seascapes, the dashed line shows National Territorial Waters (12 nm). The Plaice Box is displayed by the dotted line. Fishing intensity of 0 means that no fishing activity has been observed during the study period, or that the average fishing activity was based on < 3 separate VMS-recordings, of which the activity cannot be shown due to privacy laws. Current Natura 2000 areas are displayed, with red lines showing protected areas under de Bird Directive, pink lines showing protected areas under the Habitat Directive. Purple lines outline protected areas under both the Bird and Habitat Directive.

Fishing effort was quantified in the different seascapes (Fig 3A–3D), showing a clear separation of the main fishing grounds for each fishing gear (Table 1). Beam-Sole was mainly concentrated in seascapes 8 (Southern North Sea; 65%) and 6 (Central Oyster Grounds; 27%), while Beam-Plaice showed a preference for seascapes 5 (Dogger Bank; 63%) and 4 (Coastal Denmark; 21%). Otter-Mix had three clearly separated fishing locations, all located within seascapes 6 (Central Oyster Grounds; 62%) and 7 (Cleaver Bank; 28%). All other Dutch demersal fisheries were strongly concentrated in the coastal zone, with some additional locations in seascapes 4 and 7, showing that the three selected fisheries represented the main Dutch fisheries on the open North Sea (Fig 3D). When calculating the fisheries hotspots, we found that their spatial distribution generally captured the overall patterns of fishing activity (Fig 3E and Table 1). Beam-Plaice showed less hotspots compared with Beam-Sole and Otter-Mix, caused by yearly differences in the location of the 1% most intense fished grid cells, resulting in only a few grid cells belonging >6 years to the top 1%.

### Environmental factors

In the main fishing ground of Beam-Sole (seascapes 6 and 8), five environmental gradients from the PCA had an eigenvalue larger than 1 and were used for the further analysis (Table 2). The most important environmental gradient (PC1) in this area represents bottom temperature, depth, wave height, BSS, and distance to harbours and the nearest coastline (S20 Fig). High values reflect relative shallow, warm waters near the shore (and harbours), with high BSS and low wave action. The second environmental gradient (PC2) mostly reflects BPI at different scales, where higher values are relative elevations of the seafloor.

**Table 2:** Loadings for the relevant principal component (eigenvalue >1) from a principal component analysis of the full set of variables for the main fishing grounds of the three fisheries.

	Beam-Sole					Beam-Plaice						Otter-Mix				
Principal component	**PC1**	**PC2**	**PC3**	**PC4**	**PC5**	**PC1**	**PC2**	**PC3**	**PC4**	**PC5**	**PC6**	**PC1**	**PC2**	**PC3**	**PC4**	**PC5**
Total variance explained (%)	32.3	23.5	16.6	10.7	6.3	28.4	25.0	14.6	8.5	8.1	5.9	33.5	24.8	15.2	9.0	5.6
**Component loadings**																
Salinity (PSU)	-0.066	0.059	**0.413**	0.181	**0.342**	0.237	-0.267	-0.156	0.138	0.004	**0.357**	**-0.323**	-0.110	-0.066	-0.271	-0.267
Gravel content (%)	0.085	0.057	0.259	**-0.548**	-0.085	0.095	0.060	-0.021	**0.552**	-0.124	-0.104	-0.015	-0.032	-0.201	-0.035	-0.255
Mud content (%)	-0.192	-0.048	-0.252	-0.288	**0.444**	-0.248	-0.075	0.095	**0.311**	**0.490**	0.099	0.108	0.217	0.225	**-0.549**	0.113
Sand content (%)	0.072	-0.010	-0.025	**0.654**	-0.256	0.206	0.050	-0.086	**-0.524**	**-0.432**	-0.056	-0.108	-0.217	-0.216	**0.554**	-0.100
BPI 5km	-0.086	**-0.368**	0.091	-0.013	-0.340	-0.167	-0.004	**-0.421**	0.164	-0.274	-0.065	-0.098	**0.306**	0.093	0.245	**-0.332**
BPI 10km	-0.099	**-0.396**	0.092	-0.017	-0.308	-0.209	0.005	**-0.441**	0.174	-0.257	-0.034	-0.124	**0.364**	0.100	0.224	-0.255
BPI 30km	-0.107	**-0.429**	0.089	-0.049	-0.099	**-0.308**	0.021	**-0.342**	0.067	-0.114	0.008	-0.152	**0.398**	0.064	0.099	-0.041
BPI 50km	-0.108	**-0.430**	0.074	-0.030	0.077	**-0.346**	-0.003	-0.271	-0.135	0.104	-0.002	-0.152	**0.401**	0.017	0.057	0.051
BPI 75km	-0.109	**-0.409**	0.035	0.015	0.238	-0.289	-0.020	-0.217	-0.304	**0.305**	0.018	-0.135	**0.402**	-0.035	0.034	0.148
Depth (m)	**0.305**	-0.110	0.222	-0.044	0.081	0.227	**0.357**	0.037	0.049	-0.034	0.200	**0.305**	-0.233	-0.062	0.062	-0.207
BSS	**0.311**	0.204	-0.014	-0.093	**-0.322**	0.204	0.178	-0.174	-0.071	0.269	**-0.573**	0.177	0.138	**-0.367**	0.193	**0.424**
Maximum temperature difference (°C)	0.182	-0.133	**-0.449**	-0.072	-0.055	-0.161	**0.403**	0.097	0.017	-0.059	0.187	**0.357**	0.080	0.182	0.099	-0.145
Average bottom temperature (°C)	**0.359**	-0.145	-0.050	0.104	0.198	0.095	**0.416**	-0.102	0.117	-0.011	0.145	**0.338**	0.130	-0.148	-0.070	-0.200
Maximum bottom temperature (°C)	0.298	-0.166	-0.296	0.031	0.120	-0.094	**0.424**	0.058	0.078	-0.070	0.212	**0.367**	0.099	0.096	0.027	-0.180
Minimum bottom temperature (°C)	0.202	-0.056	**0.344**	0.232	**0.354**	0.298	-0.247	-0.218	0.080	0.155	-0.095	-0.253	0.005	**-0.365**	-0.275	-0.014
Average significant wave height (m)	**-0.345**	0.053	-0.239	0.113	0.083	**-0.301**	-0.218	0.251	-0.097	-0.137	0.157	-0.084	-0.155	**0.529**	0.015	-0.012
Maximum significant wave height (m)	**-0.324**	0.039	-0.216	0.052	0.038	**-0.335**	-0.079	**0.319**	-0.051	-0.155	-0.099	-0.044	-0.114	**0.429**	0.239	**0.409**
Distance to closest coastal line (m)	**-0.348**	0.114	0.051	0.198	0.001	0.182	-0.214	-0.221	-0.112	0.067	**0.557**	-0.283	-0.175	0.183	0.011	**-0.348**
Distance to closest Dutch harbour (m)	-0.261	0.151	**0.318**	-0.128	-0.174	0.008	-0.284	0.181	0.273	**-0.383**	-0.139	**-0.360**	-0.109	-0.036	0.051	0.186

Loadings larger than |0.3| are given in bold.

For the Beam-Plaice main fishing ground (seascapes 4 and 5), the PCA distinguished six independent main environmental gradients (Table 2). The first environmental gradient (PC1) reflects wave action and mid-scale BPI (30 and 50km), with higher values representing areas with elevations at the mid-scale and higher wave action (S21 Fig). The second environmental gradient (PC2) captures water temperature and absolute depth, with low values corresponding with deeper and colder waters.

For the Otter-Mix main fishing ground (seascapes 6 and 7), the PCA identified 5 environmental gradients (Table 2), of which the most important one (PC1) reflects salinity, depth, bottom temperature and harbour distance. This gradient ranges from saline, deep, and cold waters offshore (low values) to less saline, shallow, warmer waters nearshore (high values; S22 Fig). The second environmental gradient (PC2) represents seabed morphology, with high values indicating local depressions.

### MaxEnt distribution model

**Fig 4:**
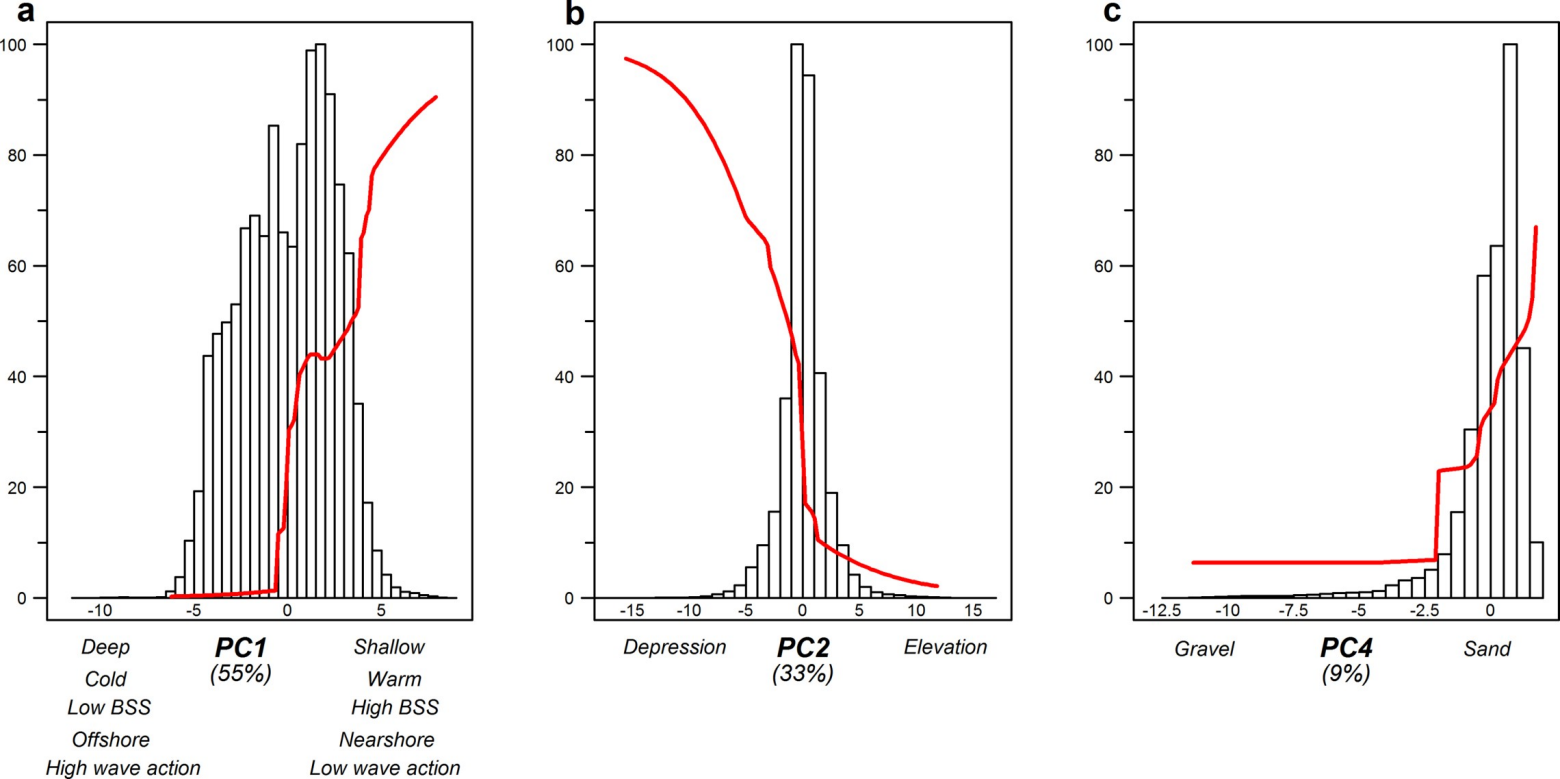
Response curves of the environmental gradients in the MaxEnt model for Beam-Sole, in relation to the abundance of the specific environmental condition. Each figure shows the representative range of a principal component (x-axis), the probability of fishing hotspot presence within that range (red line), and the abundance frequency of that range on the main fishing ground (histogram). The figures are ordered in decreasing importance for model explanation, which can be found between brackets underneath the x-axis. Only curves that explain >5% of the model are included.

Our fisheries-specific MaxEnt distribution models yielded response curves of fishing hotspot presence to all relevant environmental gradients (S1–S3 Tables) and determined the importance of each gradient. These response curves show preferences of the fishers, which were combined with estimates of gradient value commonness and rarity. This is assessed through the frequency distribution of environmental gradient values of the main fishing area. The plots therefore indicate the commonness or rarity of the preferred environmental conditions by the different fisheries. The values of these plots are also given in supplementary materials (S4–S6 Tables). The MaxEnt model for Beam-Sole (training AUC = 0.842; S23 Fig) showed that hotspot location was mainly explained by PC1 (55%), the gradient of depth, bottom temperature, BSS, wave action and distance to the coast and harbours. A preference was shown for shallow, nearshore waters characterized by higher bottom temperatures, high BSS, and low wave action (Fig 4). Within this area, the Beam-Sole targeted relative depressions (5–10 km) while avoiding gravelly sediments. These preferred environmental ranges were, except for gravel content, relatively rare in the main fishing ground.

**Fig 5:**
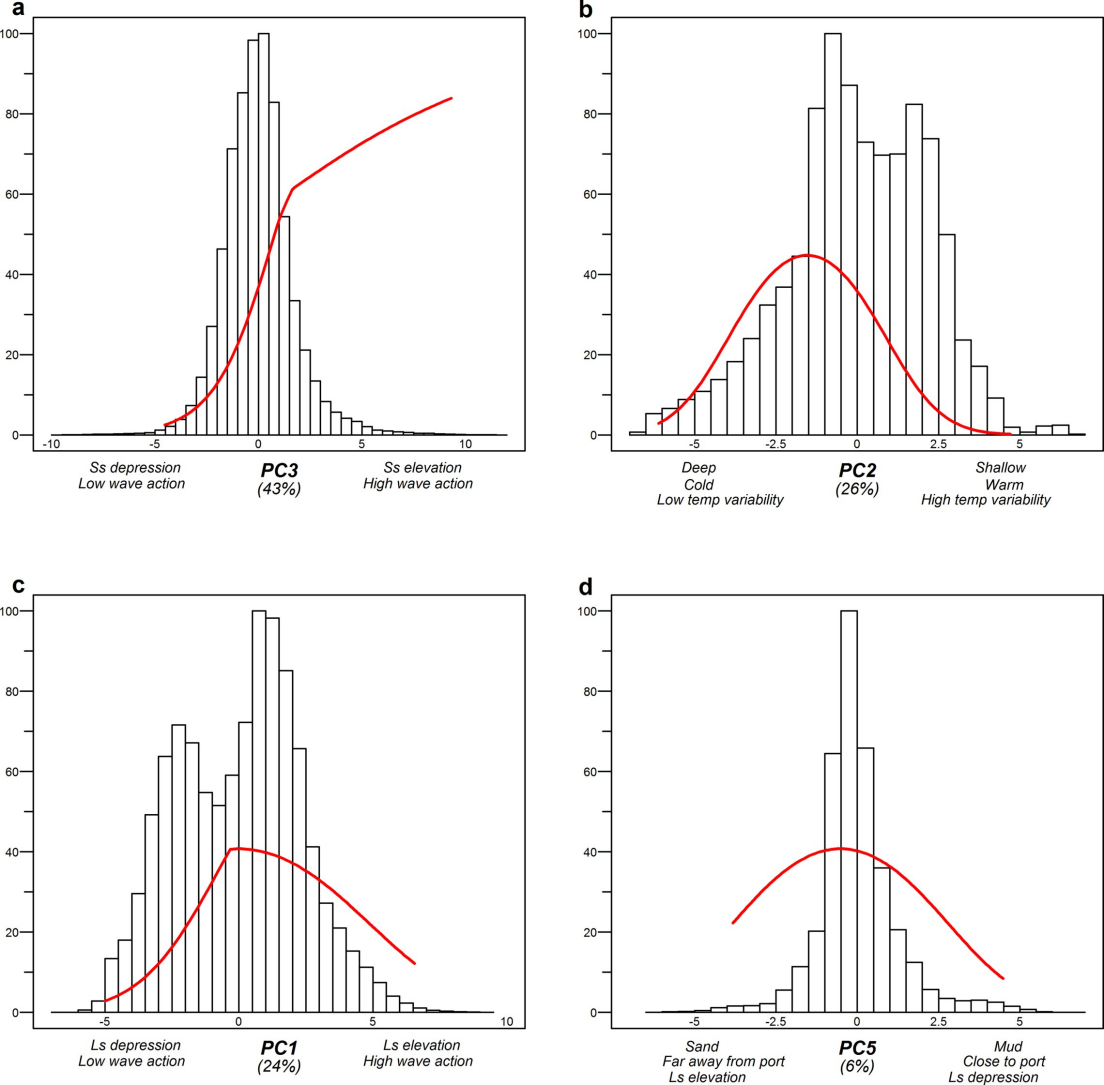
Response curves of the environmental gradients in the MaxEnt model for Beam-Plaice, in relation to the abundance of the specific environmental condition. Each figure shows the representative range of a principal component (x-axis), the probability of fishing hotspot presence within that range (red line), and the abundance frequency of that range on the main fishing ground (histogram). The figures are ordered in decreasing importance for model explanation, which can be found between brackets underneath the x-axis. Only curves that explain >5% of the model are included (Ls: Large-scale, Ss: Small-scale).

The fishing hotspots of Beam-Plaice (training AUC = 0.865; S23 Fig) were mostly explained by PC3 (43%), reflecting small-scale (5–10 km) seabed morphology and wave action, with higher probability in areas with high wave action and areas that elevate of the seabed (Fig 5). The two environmental gradients most dominant in the area (PC1 and PC2) were also important (with 24% and 26% respectively). They represented a gradient in depth, bottom temperature and temperature variability (PC2) and a gradient in wave action and large-scale morphology (PC1). For the two most important gradients (PC3 and PC2), the preferred locations were more rare habitats. Especially for the most important gradient (PC3), small-scale elevated areas with high wave action were targeted, which are a relatively rare habitat in the main fishing ground.

**Fig 6:**
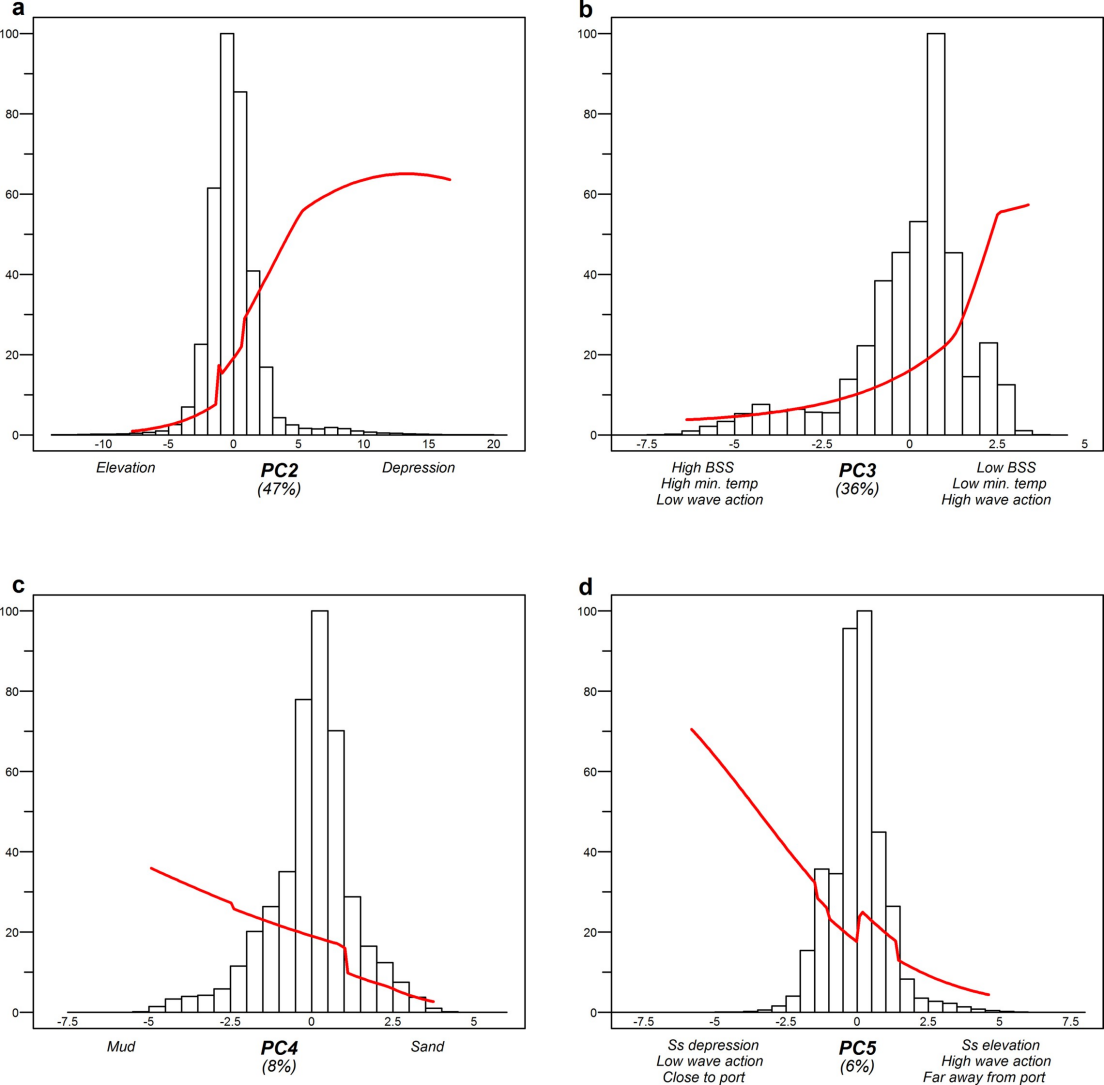
Response curves of the environmental gradients in the MaxEnt model for Otter-Mix, in relation to the abundance of the specific environmental condition. Each figure shows the representative range of a principal component (x-axis), the probability of fishing hotspot presence within that range (red line), and the abundance frequency of that range on the main fishing ground (histogram). The figures are ordered in decreasing importance for model explanation, which can be found between brackets underneath the x-axis. Only curves that explain >5% of the model are included (Ls: Large-scale, Ss: Small-scale).

The Otter-Mix hotspot locations (training AUC = 0.843; S23 Fig) were mostly explained by seabed morphology (47%), with an increase in probability at relative depressions (Fig 6). In addition, Otter-Mix showed a preference for areas with low bed shear stress, low minimum temperatures and high wave actions (36%). The presence of these hotspots was somewhat affected by sediment type (8%), with a preference towards muddy sediments. Within the Otter-Mix fishing ground, the preferred areas with these conditions were relatively rare.

## Discussion

We confirmed our hypothesis that stable fishing hotspot locations are associated by highly specific environmental conditions, showing that fishers have a clear preference for particular seabed habitats. The majority of these targeted habitats are uncommon in their fishing grounds. Beam-Sole hotspots were primarily targeting depressions within shallow, nearshore waters, with high bottom temperatures, high BSS values, and low wave action. Beam-Plaice was concentrated at small-scale ridges (5-10km) subjected to high wave action, which were located on top of large-scale elevations around a depth of -35 meters. Otter-Mix targeted relative depressions with low values of BSS, low minimum temperatures, high wave action and high mud content. Our results emphasize that, from a conservation perspective, calculations of fishing efforts (annual % of the area fished) should be performed for each specific habitat separately, instead of for the whole North Sea. We show that particular habitats are subjected to much higher fishing efforts than others, while knowledge on the ecological potential of the highly preferred areas remains limited. Remarkably, part of the stable fishing hotspots is located within Natura 2000 areas, which represent the present habitat protection measures.

Fishing vessels are satellite-tracked by both the Vessel Monitoring System and the Automatic Identification System (AIS), and both data sources can be used to study fishing distributions [44]. Whereas VMS yields information on a 2-hour interval, the AIS-data has a much higher temporal resolution [45]. Complete implementation of AIS, however, is only well developed since 2012, limiting the ability to study long-term stable patterns. As the temporal resolution required for this analysis was well met by VMS data, we identified the stable fishing hotspots with VMS data solely. Our methodology of stable fishing hotspots identification comes with some other limitations as well, but we assume that our analysis is indicative of general patterns. For example, fisheries are not entirely restricted to these hotspots, with the majority of the fishing activity actually taking place outside the fisheries hotspots in most years. Especially Beam-Plaice hotspots represent only a minority of the average fishing activity, because the spatial distribution of the most intense fished areas is very variable between years. This indicates that plaice fisheries patterns are spatially different between years, potentially because plaice is relatively evenly distributed over a larger area and multiple habitats, or is characterised by opportunistic behaviour where habitat preference varies from year to year. Secondly, due to our strict definition of stable fisheries hotspots, such temporal and transient hotspots are most likely not included. Likewise, recent shifts in distribution of the sole fisheries due to the introduction of the pulse fishing technique that affect the catch efficiency and selectivity are not taken into account [46–48]. However, the hotspot definition used in this study provides a very robust estimate of stable locations subjected to the highest fishing intensities per unit area. Our MaxEnt predicted maps and the average fishing activity patterns are very similar, strengthening the assumption that results presented in this study may be interpreted as general patterns within the distinct fisheries.

Our study assumes that the fishing hotspots reflect high abundances of the different target species, potentially reflecting an idea free distribution of predators competing for an aggregated prey [49]. We found that all three fisheries prefer areas with conditions that are similar to preferred habitats of their target species. Beam-Sole fishers target warmer dynamic and sandy habitats, conditions that sole seems to prefer as well [50–53]. We show that, within these waters, Beam-Sole targets depressions. These troughs are associated with higher benthic species abundance [28,29], which may attract sole [54,55]. Beam-Plaice hotspots coincide with the Tail End of the Dogger Bank (the narrowest part at the Northeast of the Dogger Bank), a key topographical feature in plaice distribution [56]. This area mainly consist of sandy substrates, which seem preferred by plaice [53,57–59]. Moreover, the targeted area in general has higher abundances of other species [58], including some high-value by-catch species (like turbot (*Scopthalmus maxima*), brill (*Scopthalmus rhombus*) and lemon sole (*Microstomus kitt*)) in the plaice fisheries [37,38,60]. Hotspots of Otter-Mix are located in deep, muddy areas; conditions preferred by Norway lobster [26,61]. The good correspondence between hotspots and knowledge on habitat preferences of the target species make it likely that the observed fishing hotspots reflect high local densities of the specific target species.

These fisheries hotspots may be favourable for more species than the target species only, promoting rich benthic communities. The environmental variables studied here are known to strongly determine the species composition of benthic communities [32]. The troughs in between relatively stable sand ridges [62] targeted by Beam-Sole, for instance, show higher benthic species abundance and richness in the troughs than on the crests [29], possibly due to local differences in sediment content [28]. Similarly, the hotspot locations of Otter-Mix are located in the Central Oyster Grounds and Frisian Front, which score high for various benthos biodiversity metrics [63]. Both observations suggest that the conditions prevailing at the hotspot locations support rich benthic communities. Moreover, the fact that there are only a few locations with these conditions in the North Sea indicates that the species depending on these conditions probably are relatively uncommon.

Our findings that stable fishing hotspots are highly structured by environmental conditions is important for sustainable fisheries management. Bottom trawling is among the most disturbing factors of the seabed worldwide [8], but current management is dominantly directed on the limitation of both Total Allowable Catch (TACs) and average fishing effort (days-at-sea) [64]. Despite separate legislations to protect benthic habitats (the Natura 2000 network as part of the Habitat [65] and Bird [66] Directive and the Marine Strategy Framework Directive [67], for instance), hardly any regulation exist for the spatial distribution of fishing effort. Currently implied habitat protecting measures, like the Natura 2000 areas, account to some extent for habitat variability. However, the discussion on the allowed fishing practices within these areas is far from settled. We show that part of the stable fishing hotspots is located within the Natura 2000 areas, areas designated for demersal habitat protection. Hence, present fisheries management ignores that bottom trawling is highly aggregated [3] and impacts habitats differently [4]. We show that the North Sea comprises a strong, relatively fine-scale variety of demersal habitats. Moreover, we demonstrate that fishers are aware of these conditions and target very specific habitats which are uncommon within their main fishing grounds. These results prove that, in addition to the variability of physical impact, the aggregated structure of demersal fisheries affects benthic habitats and associated communities unequally. We show that rare habitats and communities are subjected to high exploitation rates, while the more common habitats and communities receive relative little fishing activity. These observations emphasize that sustainable management of benthic habitats can only be achieved if the spatial distribution of both the benthic habitats and their users are incorporated in fisheries management.

This study therefore can provide scientific underpinnings for stable fisheries hotspot distributions, and enables managers and policy makers to improve monitoring and conservation planning. Moreover, our results may be used in risk assessments of anthropogenic disturbances to specific benthic habitats and communities in the North Sea. Based on the three dominant Dutch demersal fisheries, our results emphasize that marine policy should include the heterogeneity of the North Sea and the aggregated patterns of its users to enable sustainable exploitation and sufficient nature protection.

## Supporting information

S1 Fig Absolute water depth of the study area. (TIFF)

S2 Fig Bathymetry Position Index with a radius of 5 km. (TIFF)

S3 Fig Bathymetry Position Index with a radius of 10 km. (TIFF)

S4 Fig Bathymetry Position Index with a radius of 30 km. (TIFF)

S5 Fig Bathymetry Position Index with a radius of 50 km. (TIFF)

S6 Fig Bathymetry Position Index with a radius of 75 km. (TIFF)

S7 Fig Percentage of the gravel content in the sediment. (TIFF)

S8 Fig Percentage of the mud content in the sediment. (TIFF)

S9 Fig Percentage of the sand content in the sediment. (TIFF)

S10 Fig Tidal component of the Bed Shear Stress. (TIFF)

S11 Fig Maximum modelled annual wave height. (TIFF)

S12 Fig Average modelled wave height. (TIFF)

S13 Fig Salinity. (TIFF)

S14 Fig Averaged modelled bottom temperature. (TIFF)

S15 Fig Maximum modelled bottom temperature. (TIFF)

S16 Fig Minimum modelled bottom temperature. (TIFF)

S17 Fig Maximum difference in modelled bottom temperature. (TIFF)

S18 Fig Distance to the nearest coastal line. (TIFF)

S19 Fig Distance to the nearest Dutch harbour with auction. (TIFF)

S20 Fig Visual interpretation of the relevant principal components (eigenvalue >1) on the Beam-Sole main fishing grounds. (TIFF)

S21 Fig Visual interpretation of the relevant principal components (eigenvalue >1) on the Beam-Plaice main fishing grounds. (TIFF)

S22 Fig Visual interpretation of the relevant principal components (eigenvalue >1) on the Otter-Mix main fishing grounds. (TIFF)

S23 Fig ROC-curves for the MaxEnt models for (a) Beam-Sole, (b) Beam-Plaice, and (c) Otter-Mix.
(TIFF)

S1 Table Contribution and importance of each relevant Principal Component to the Beam-Sole MaxEnt model. (DOCX)

S2 Table Contribution and importance of each relevant Principal Component to the Beam-Plaice MaxEnt model. (DOCX)

S3 Table Contribution and importance of each relevant Principal Component to the Otter-Mix MaxEnt model. (DOCX)

S4 Table Response curves of the environmental gradients in the MaxEnt model for Beam-Sole, in relation to the abundance of the specific environmental condition. (DOCX)

S5 Table Response curves of the environmental gradients in the MaxEnt model for Beam-Plaice, in relation to the abundance of the specific environmental condition. (DOCX)

S6 Table Response curves of the environmental gradients in the MaxEnt model for Otter-Mix, in relation to the abundance of the specific environmental condition. (DOCX)
