# Supplementary figures and images for: North Sea demersal fisheries prefer specific benthic habitats
Source: PLoS One. 2018 Dec 18;13(12):e0208338. doi: 10.1371/journal.pone.0208338 (PMC6298764; doi:10.1371/journal.pone.0208338)

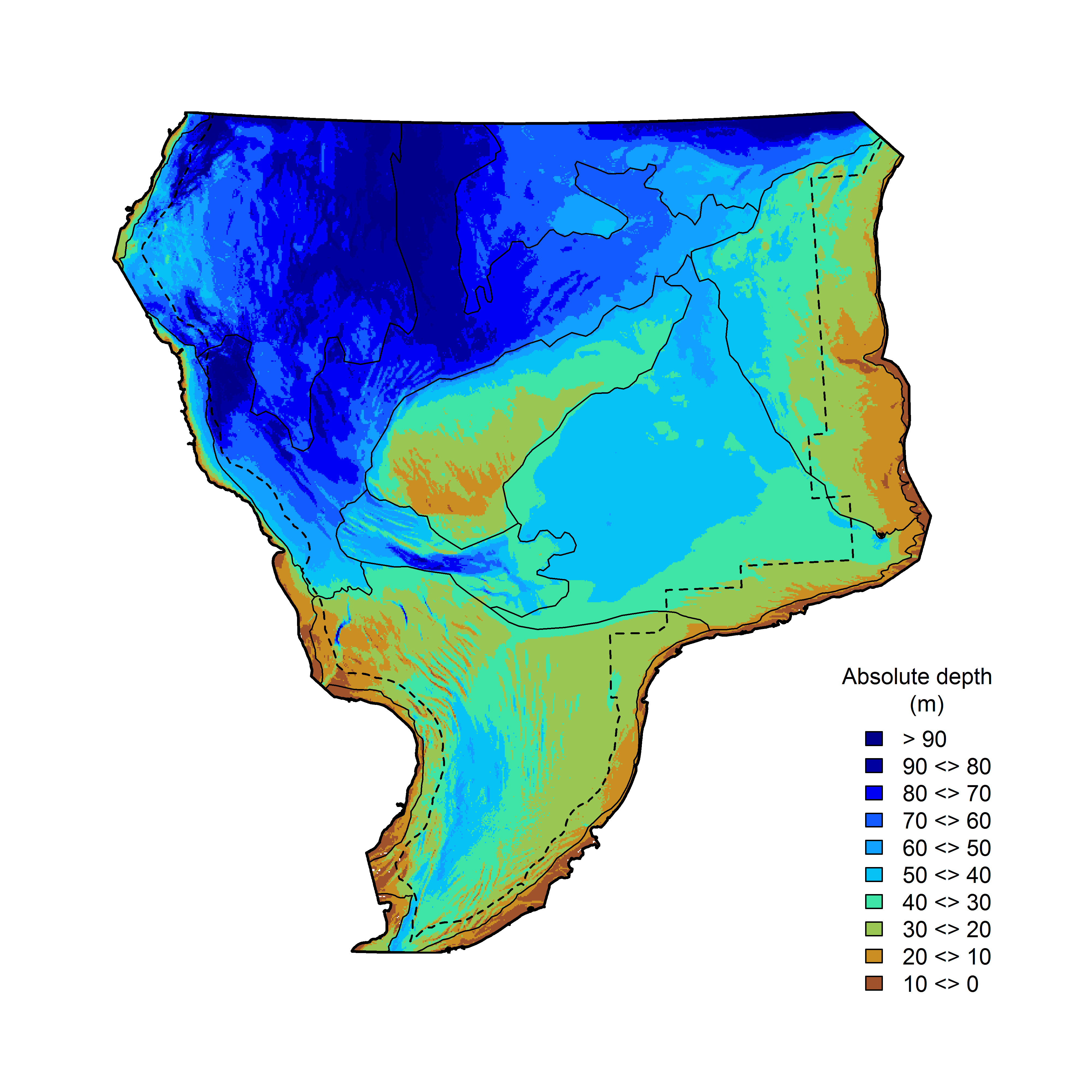

Supplement: S1 Fig — (TIFF) [file pone.0208338.s001.tiff]

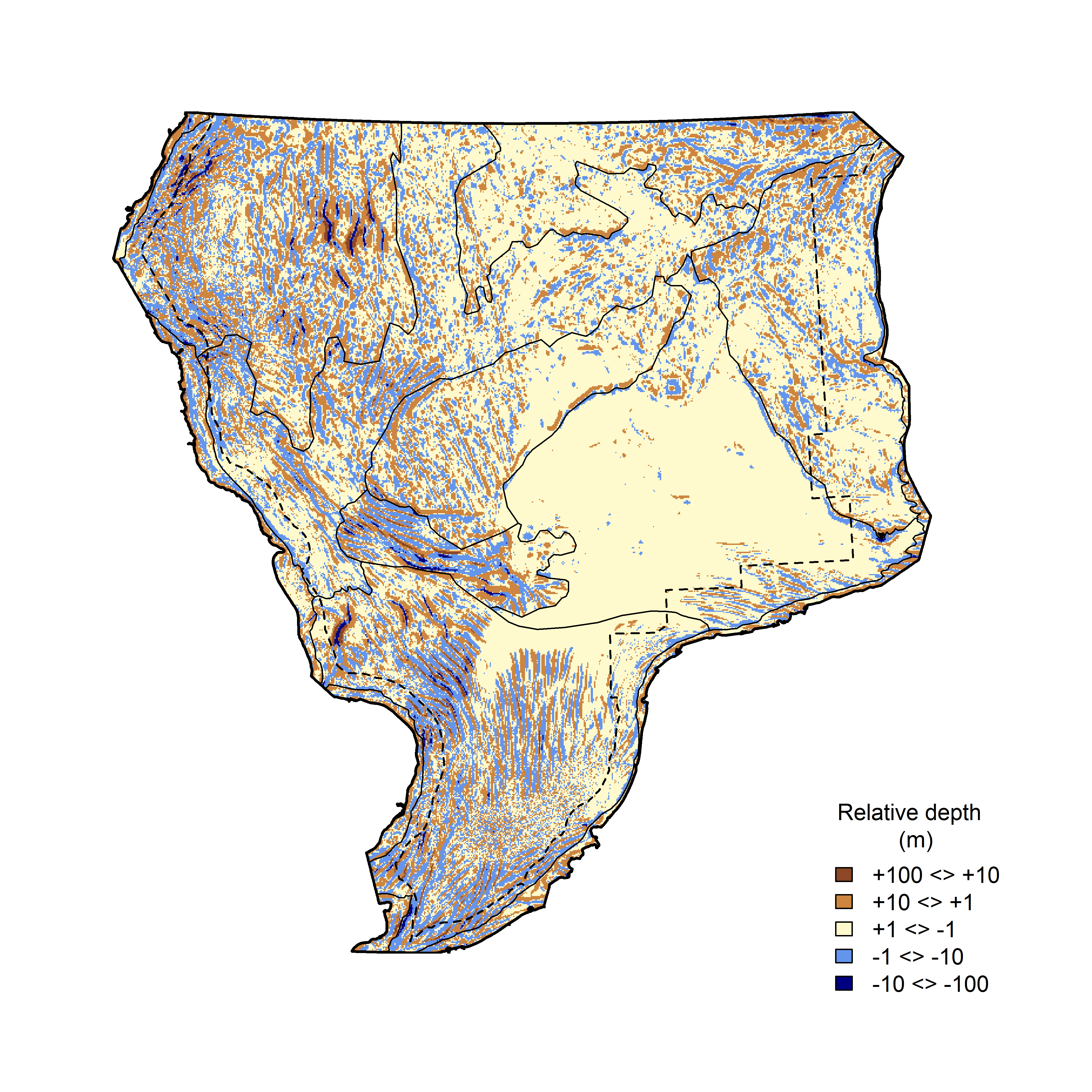

Supplement: S2 Fig — (TIFF) [file pone.0208338.s002.tiff]

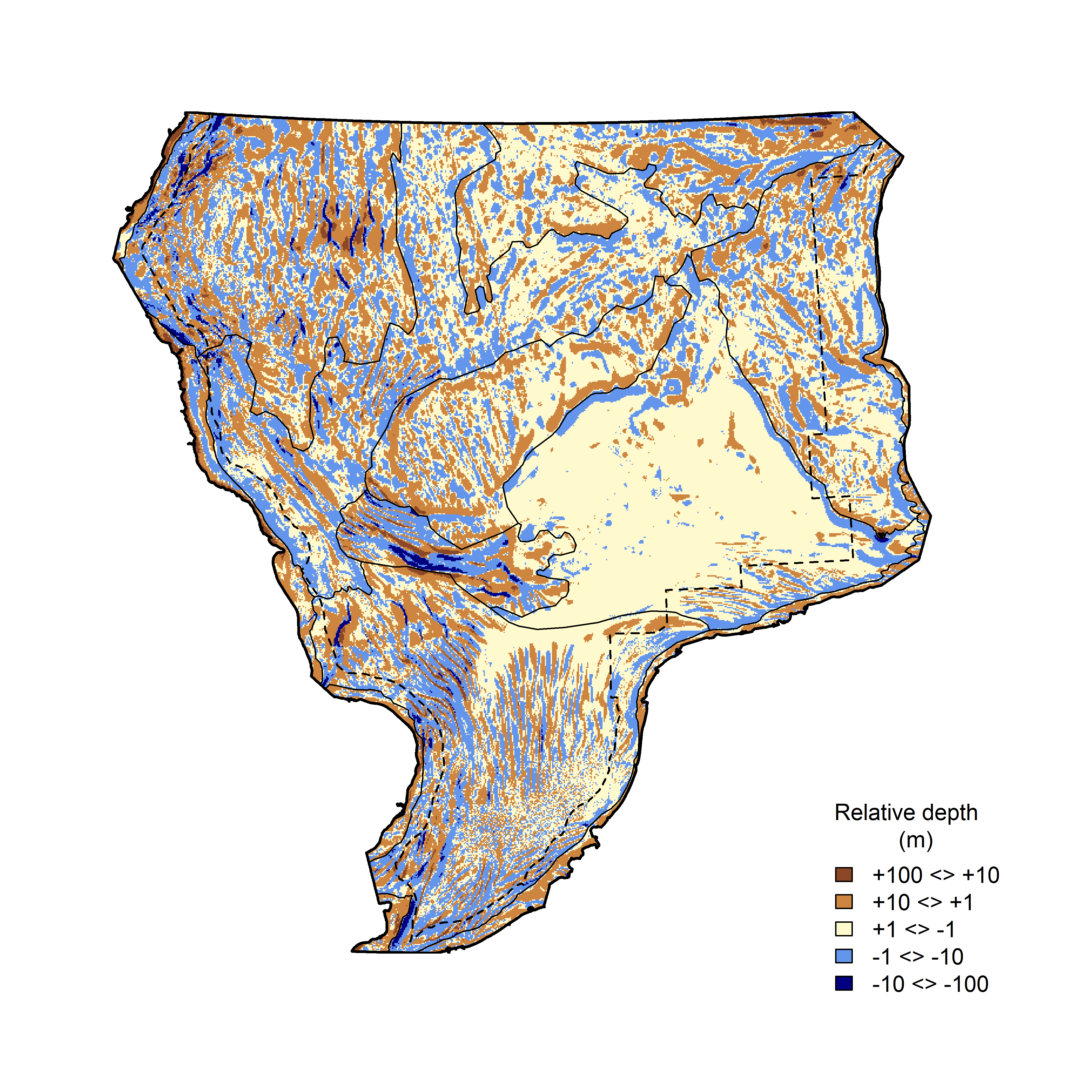

Supplement: S3 Fig — (TIFF) [file pone.0208338.s003.tiff]

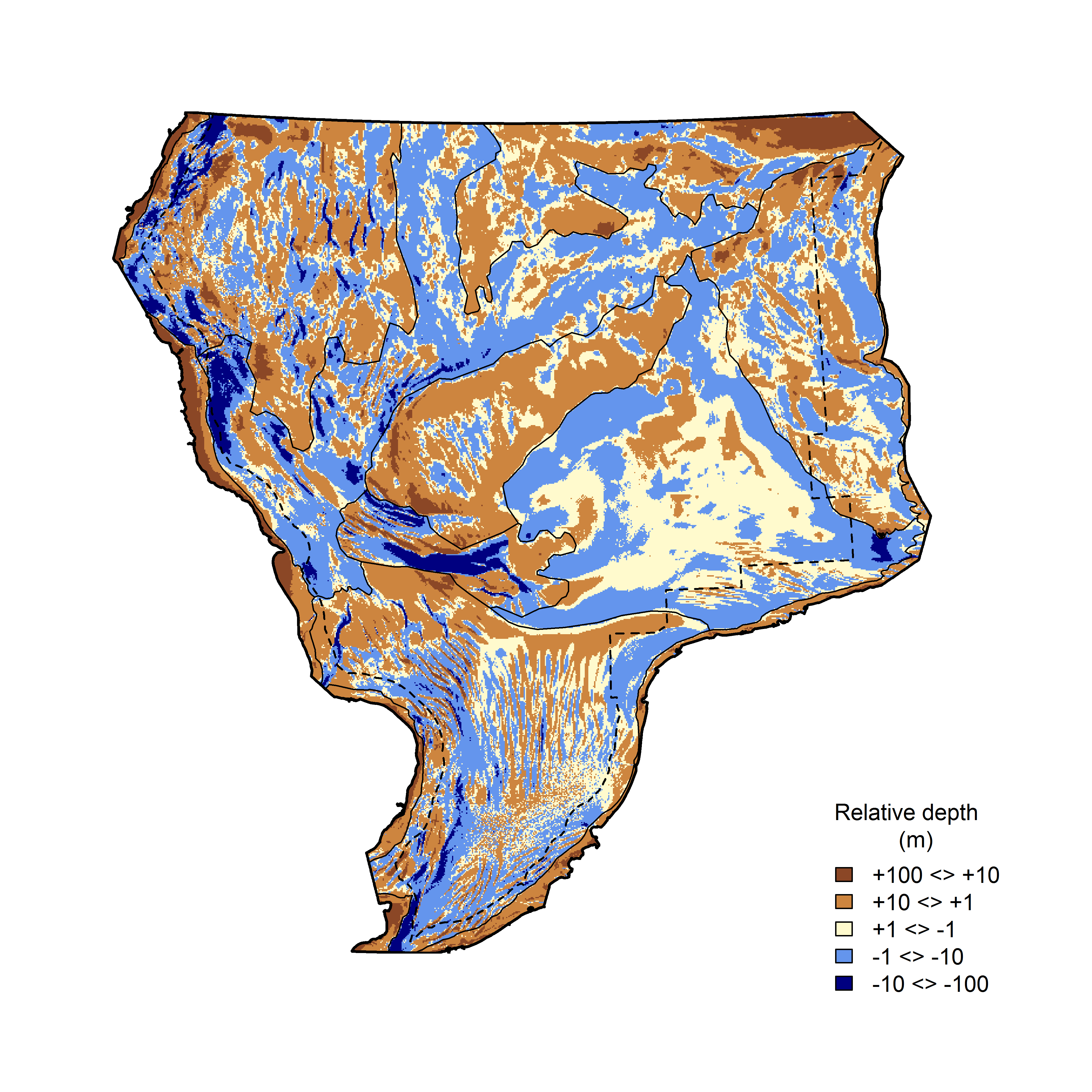

Supplement: S4 Fig — (TIFF) [file pone.0208338.s004.tiff]

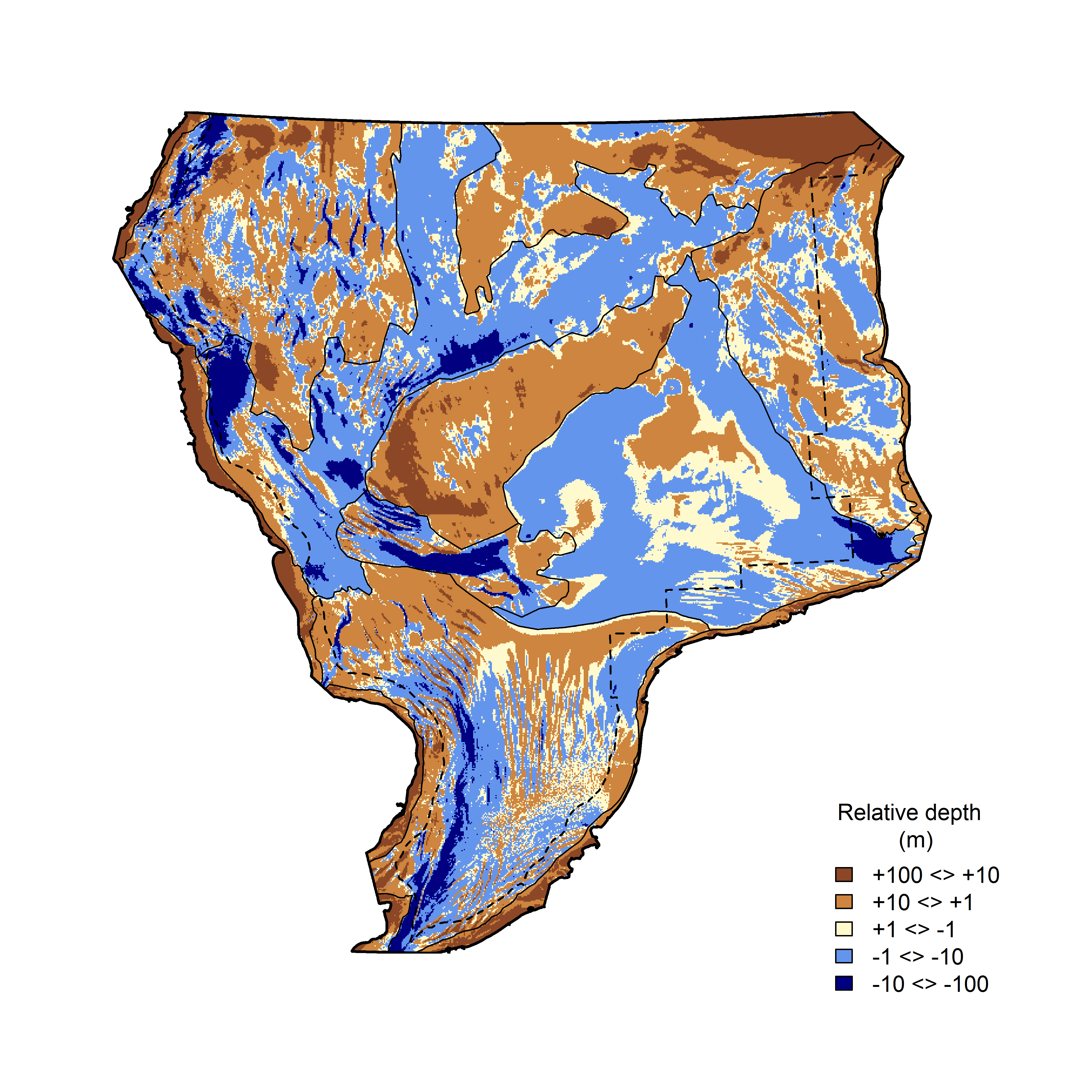

Supplement: S5 Fig — (TIFF) [file pone.0208338.s005.tiff]

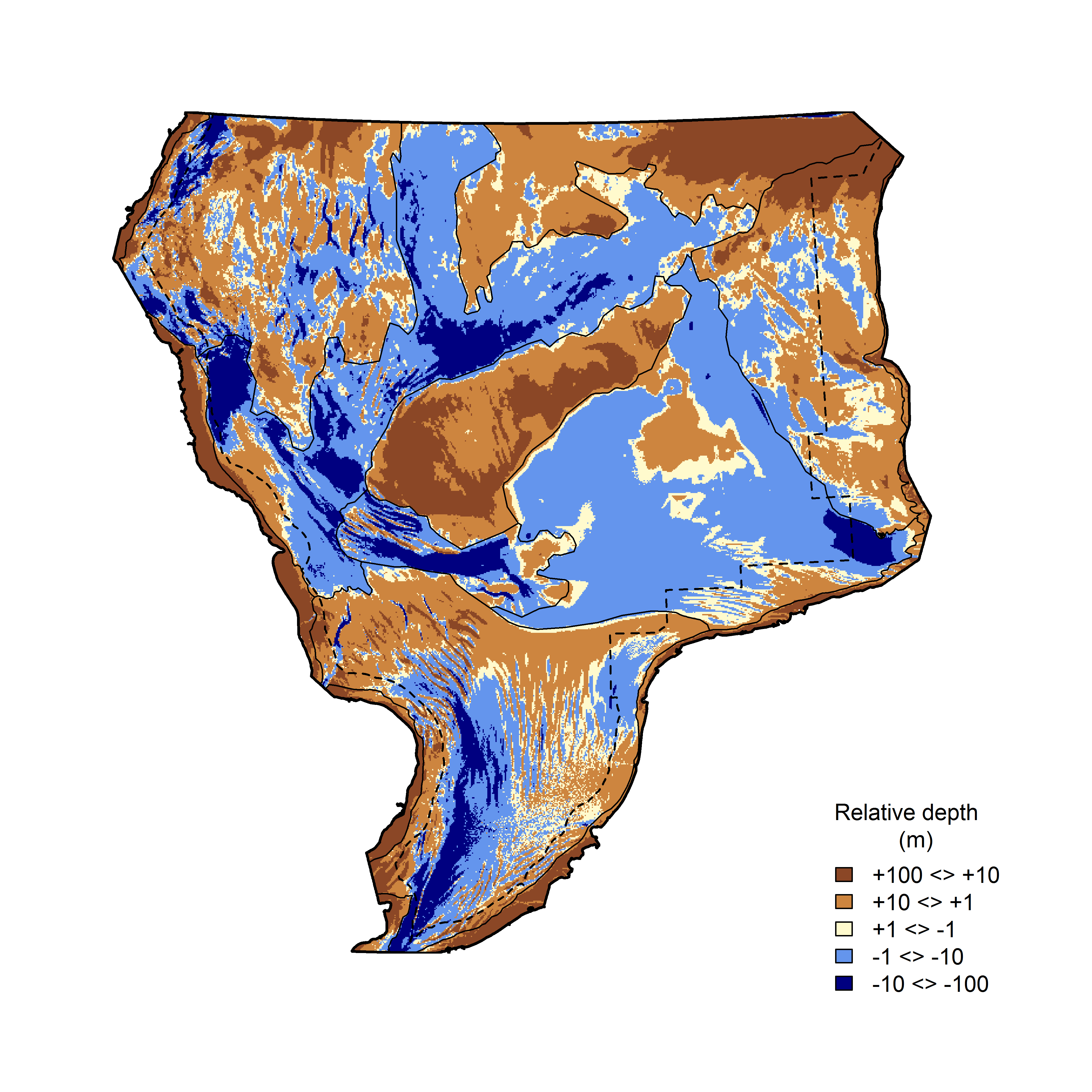

Supplement: S6 Fig — (TIFF) [file pone.0208338.s006.tiff]

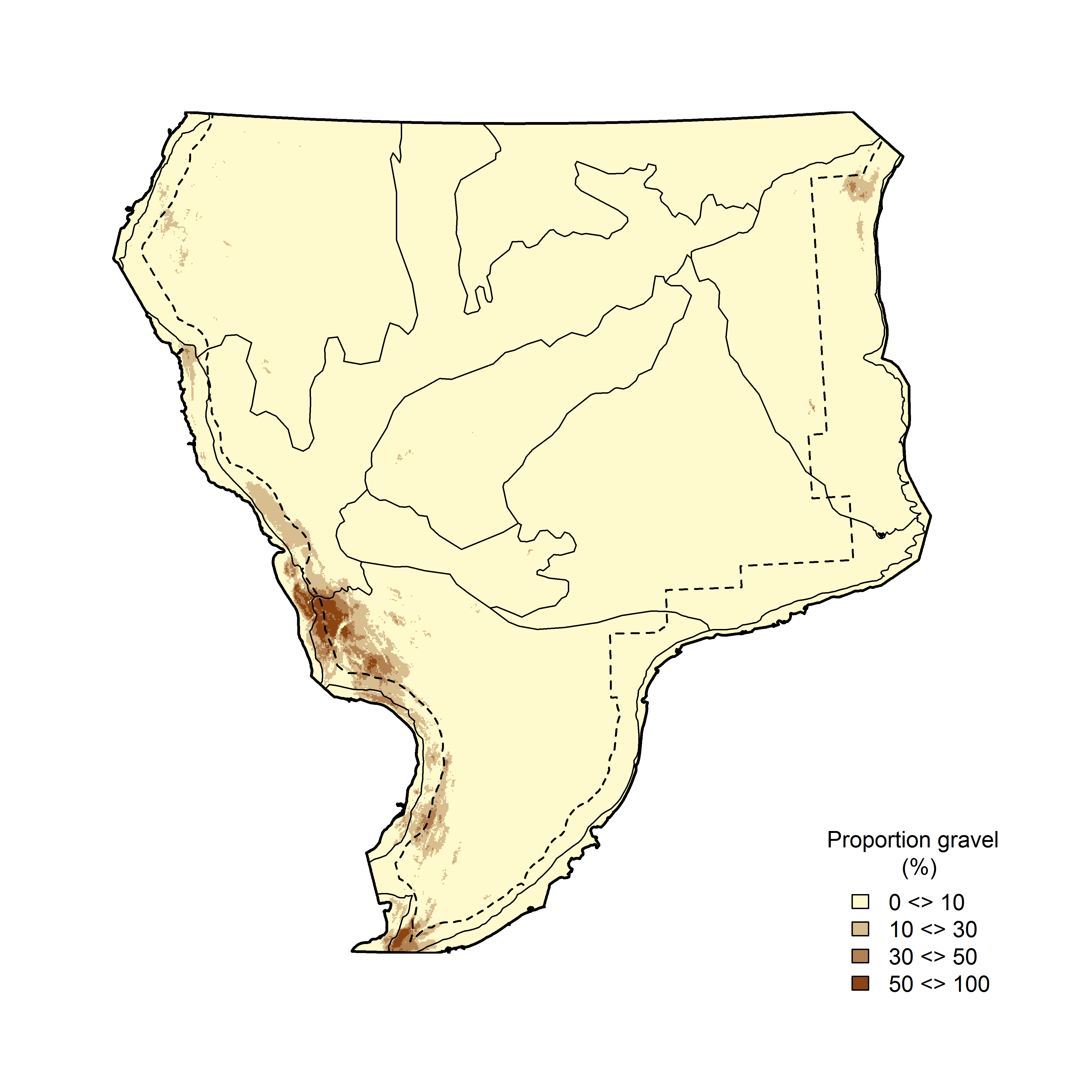

Supplement: S7 Fig — (TIFF) [file pone.0208338.s007.tiff]

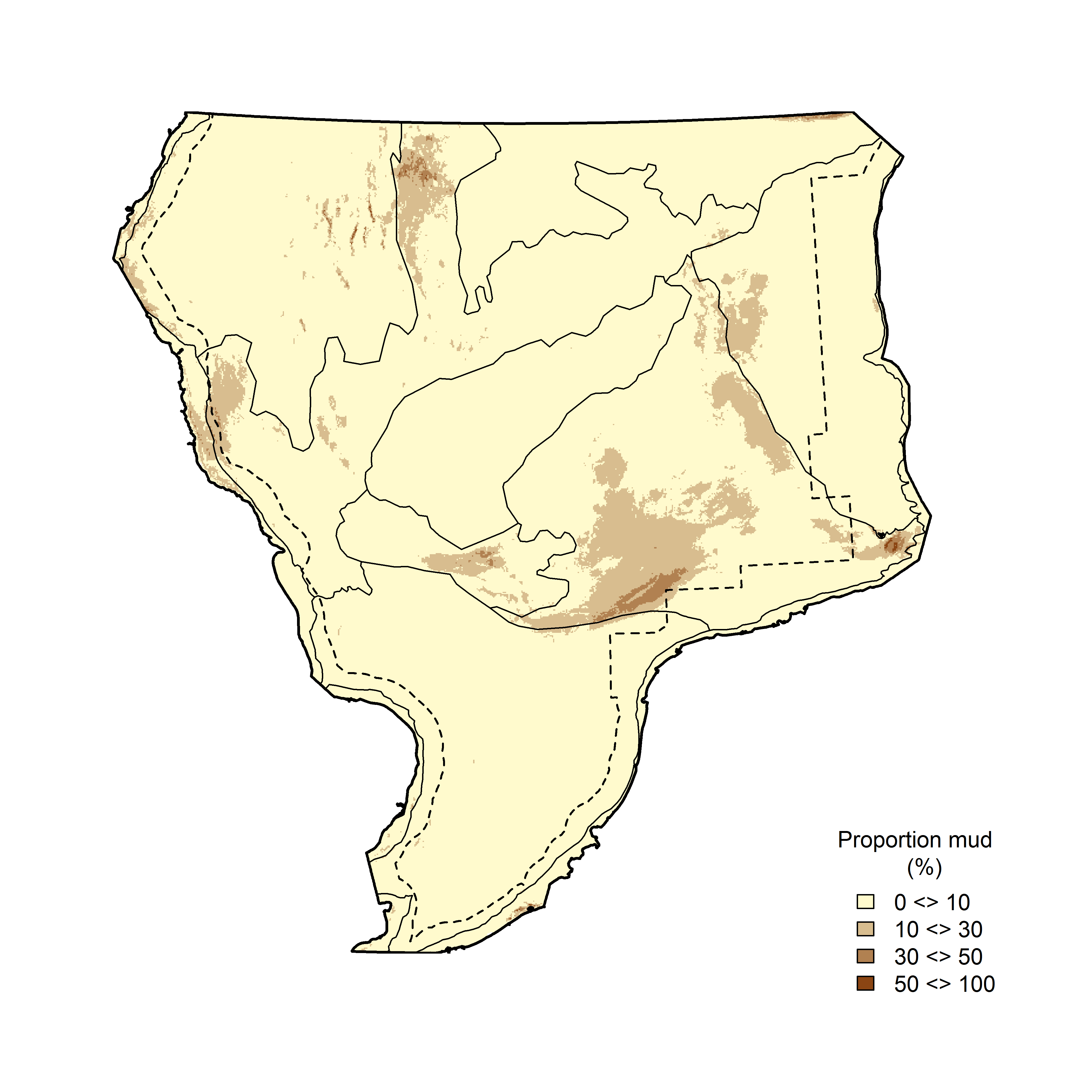

Supplement: S8 Fig — (TIFF) [file pone.0208338.s008.tiff]

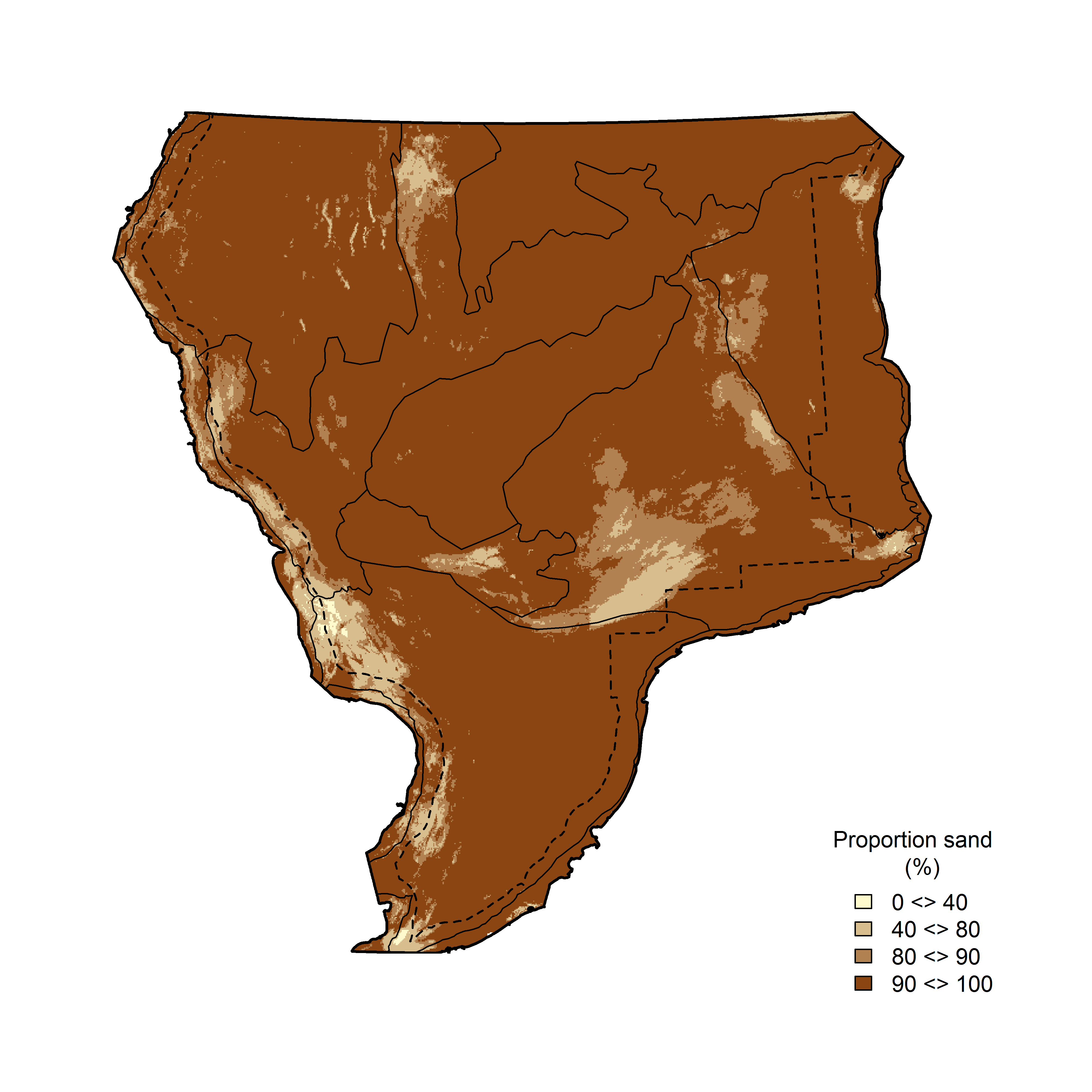

Supplement: S9 Fig — (TIFF) [file pone.0208338.s009.tiff]

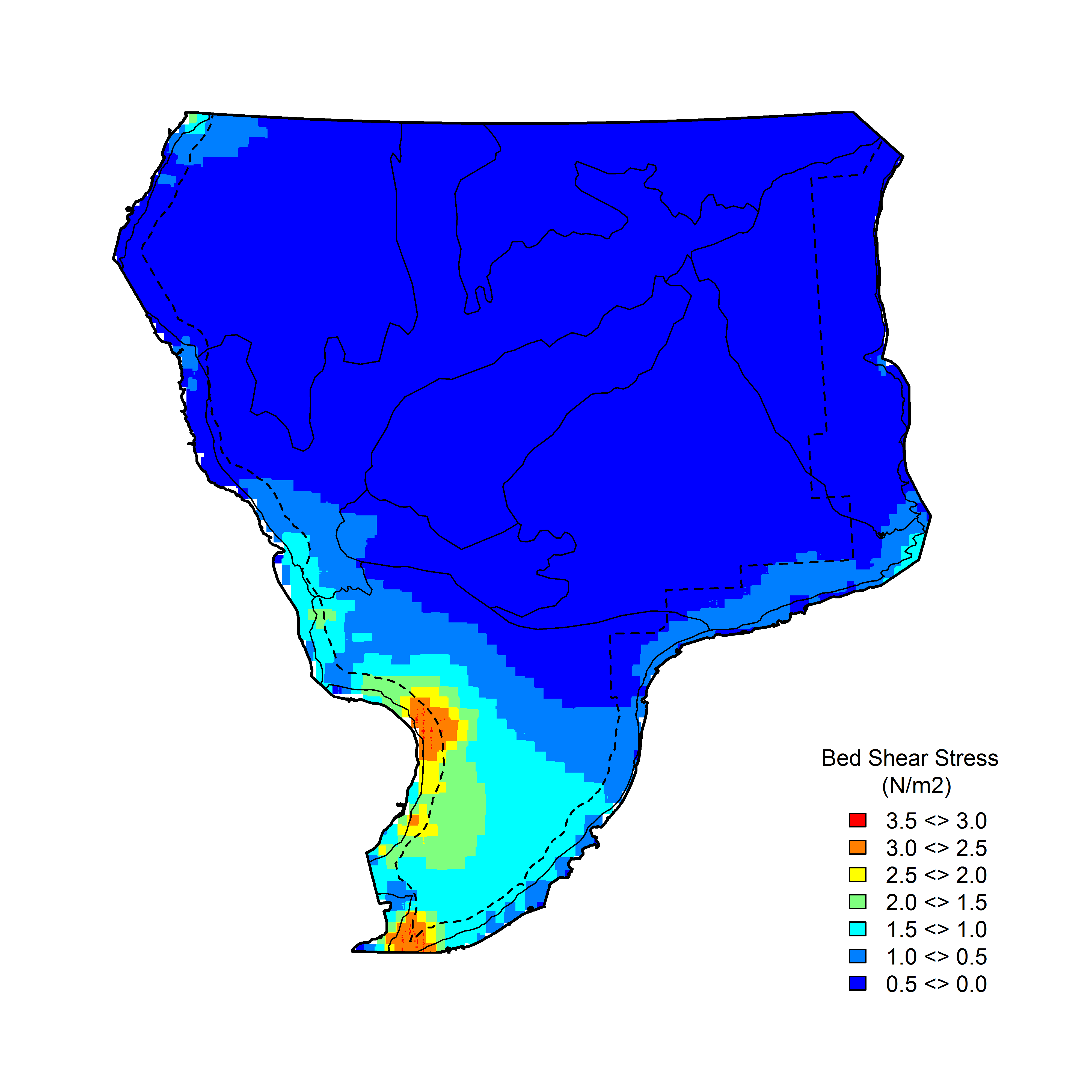

Supplement: S10 Fig — (TIFF) [file pone.0208338.s010.tiff]

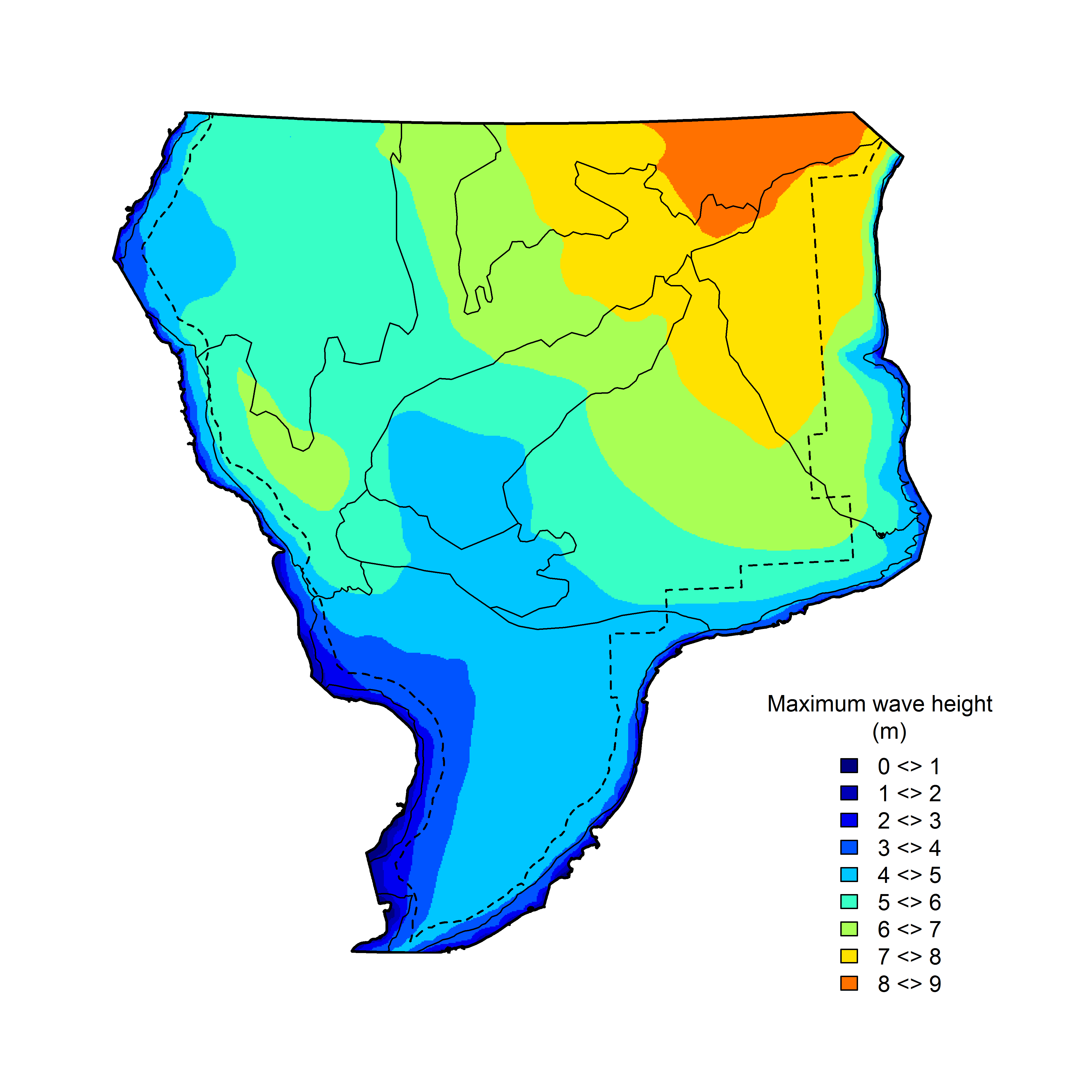

Supplement: S11 Fig — (TIFF) [file pone.0208338.s011.tiff]

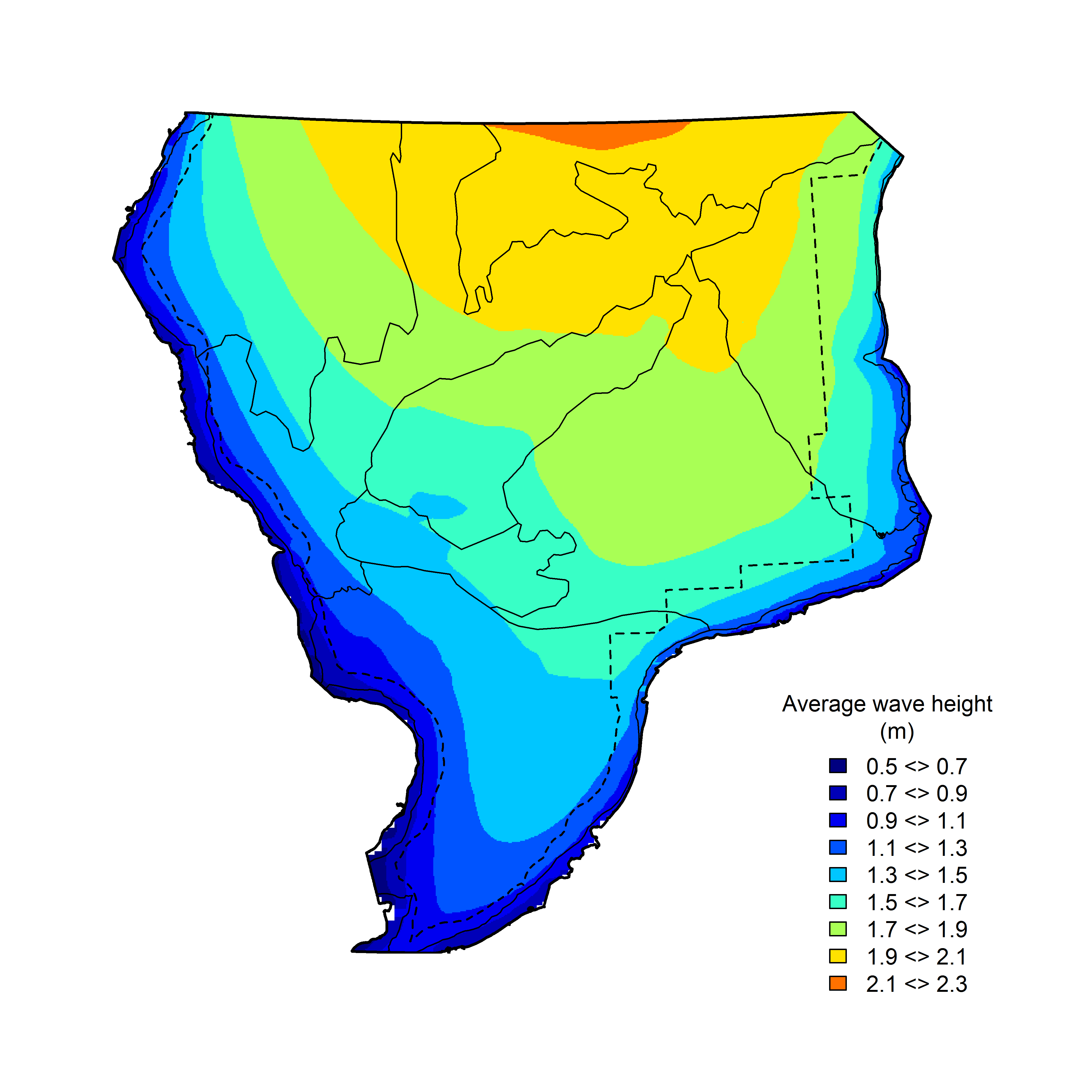

Supplement: S12 Fig — (TIFF) [file pone.0208338.s012.tiff]

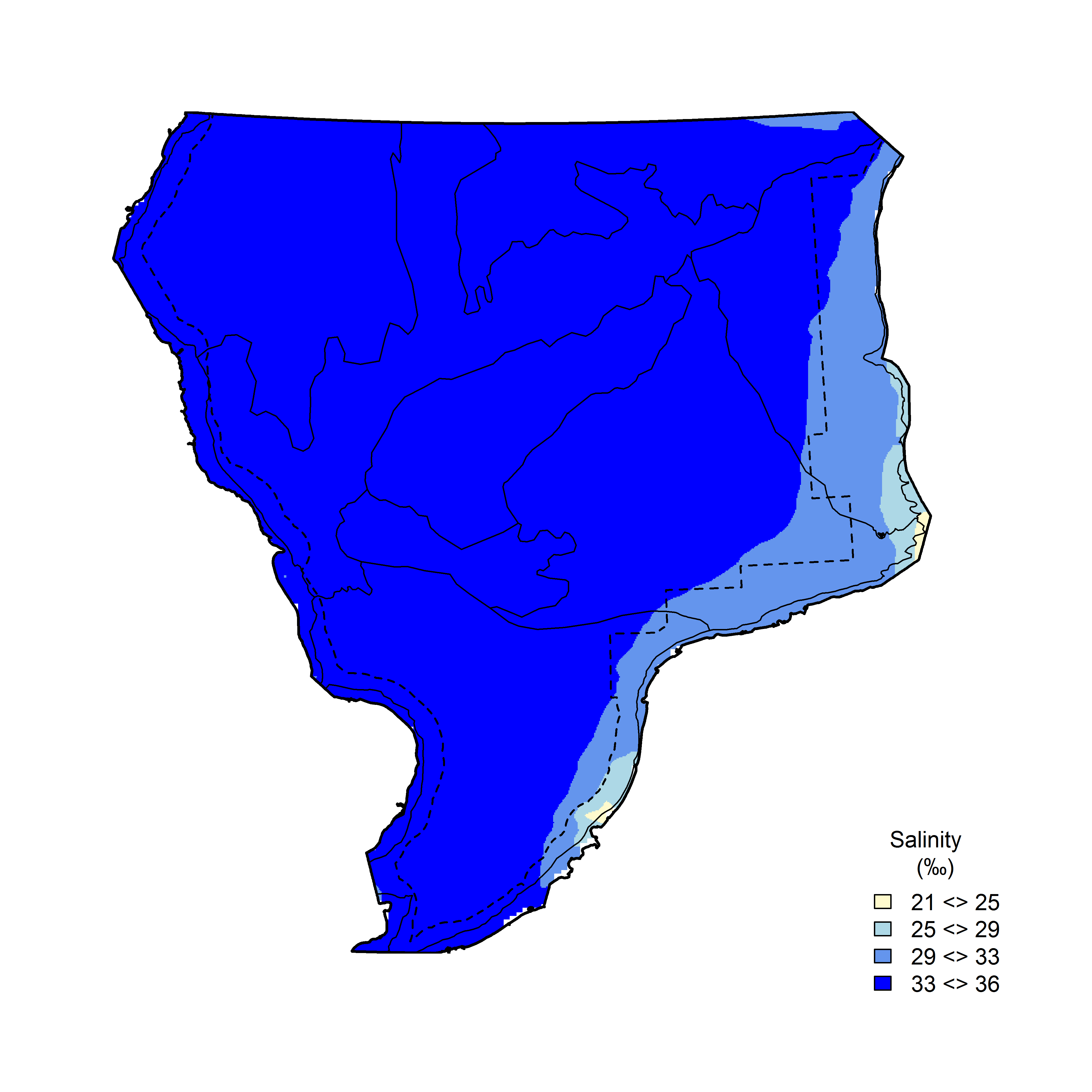

Supplement: S13 Fig — (TIFF) [file pone.0208338.s013.tiff]

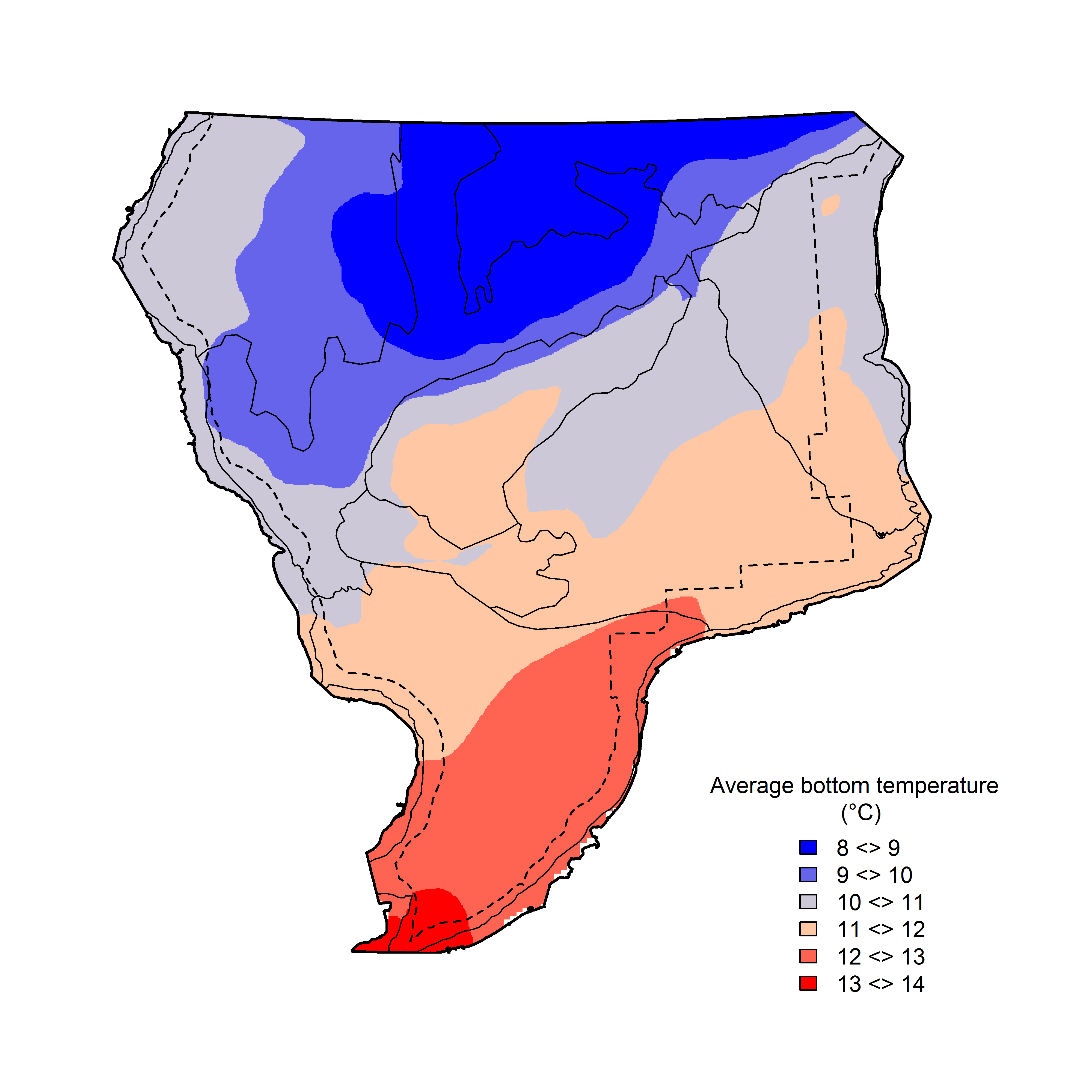

Supplement: S14 Fig — (TIFF) [file pone.0208338.s014.tiff]

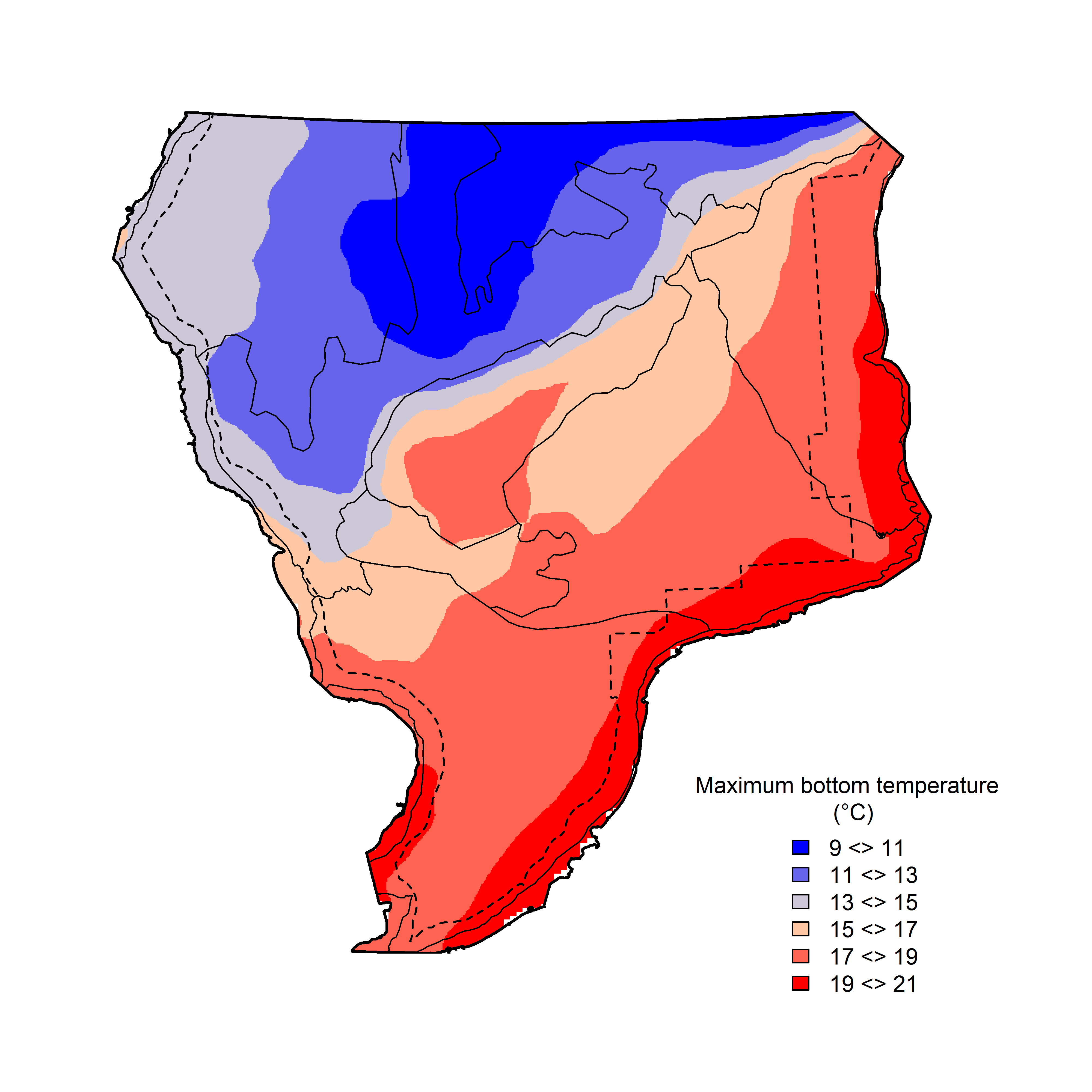

Supplement: S15 Fig — (TIFF) [file pone.0208338.s015.tiff]

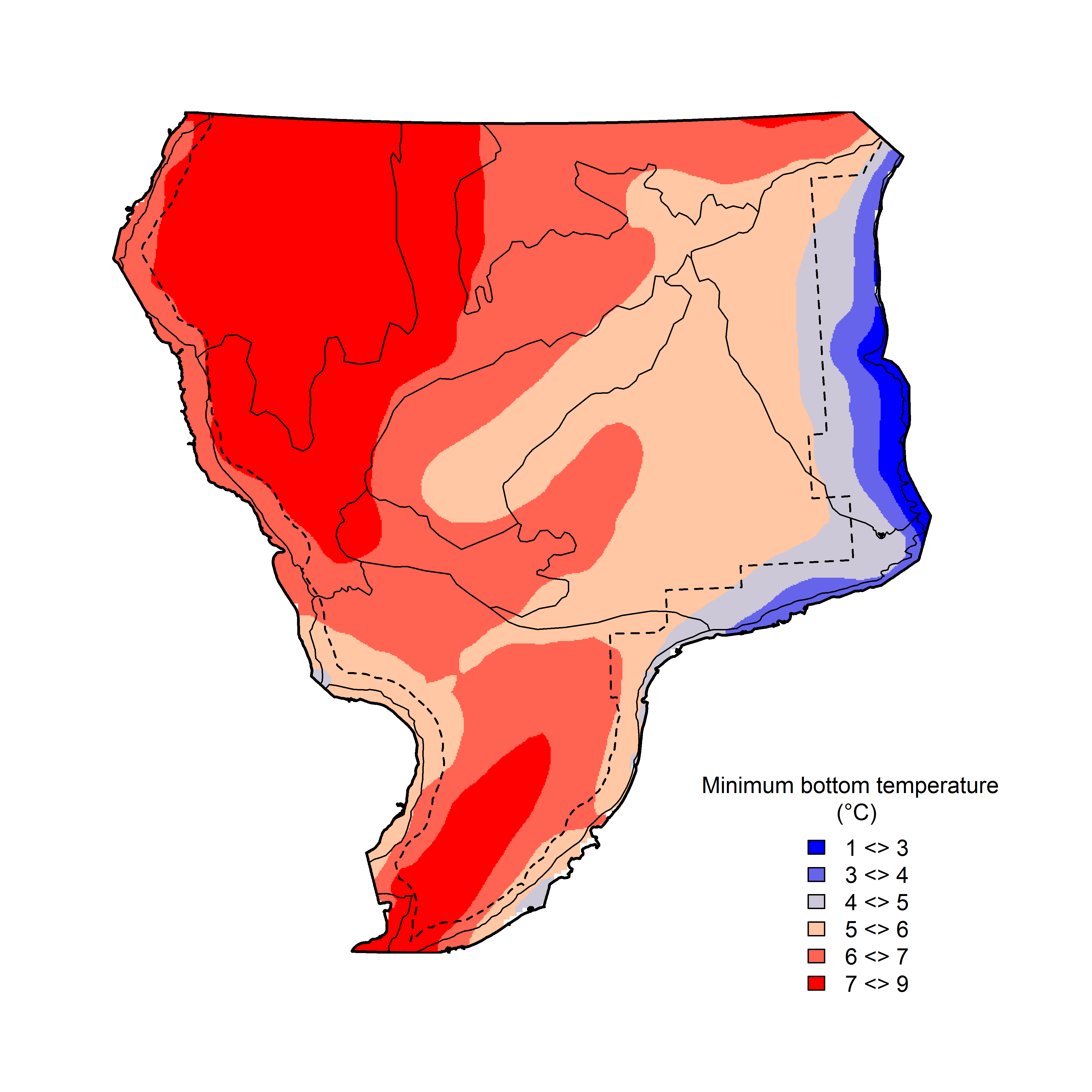

Supplement: S16 Fig — (TIFF) [file pone.0208338.s016.tiff]

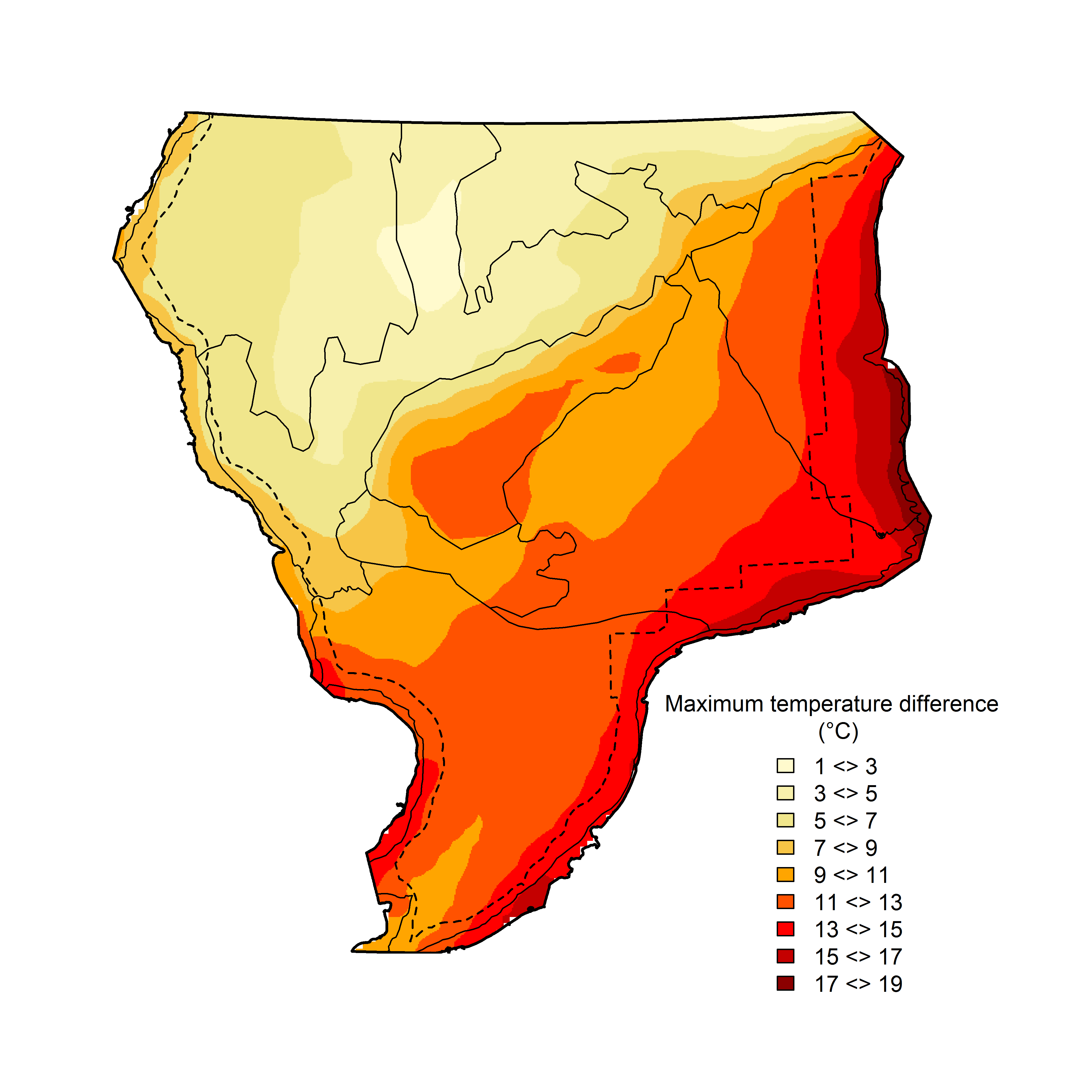

Supplement: S17 Fig — (TIFF) [file pone.0208338.s017.tiff]

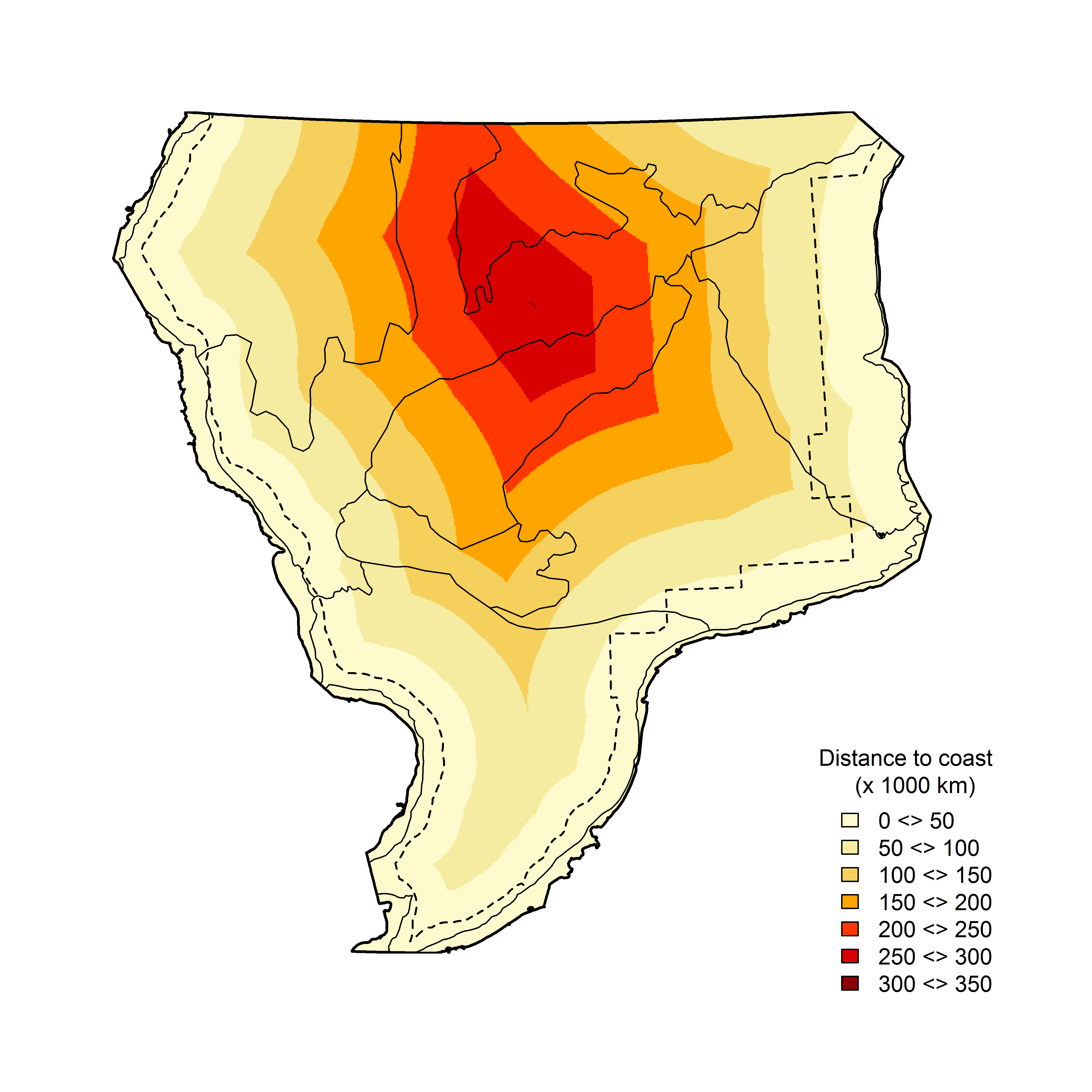

Supplement: S18 Fig — (TIFF) [file pone.0208338.s018.tiff]

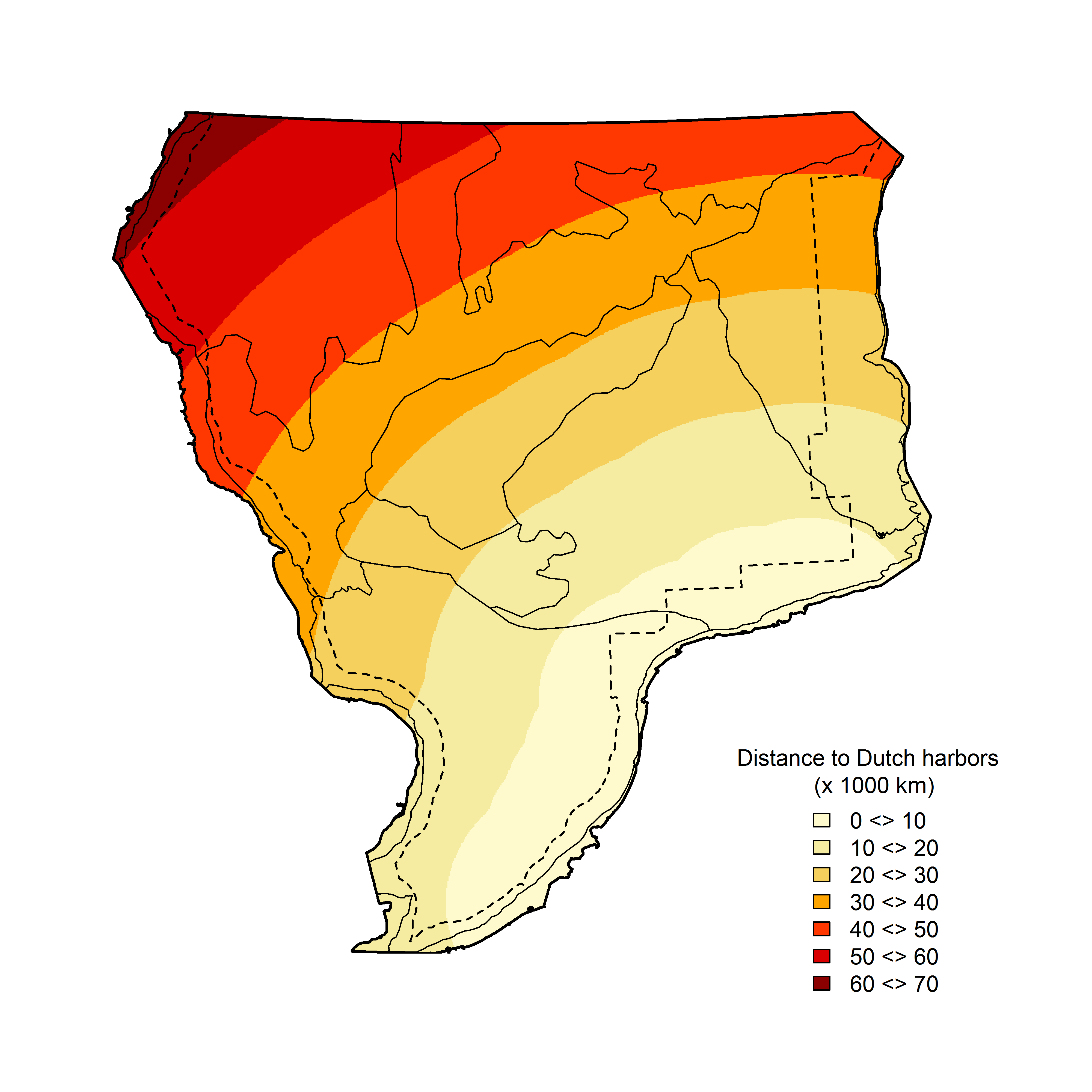

Supplement: S19 Fig — (TIFF) [file pone.0208338.s019.tiff]

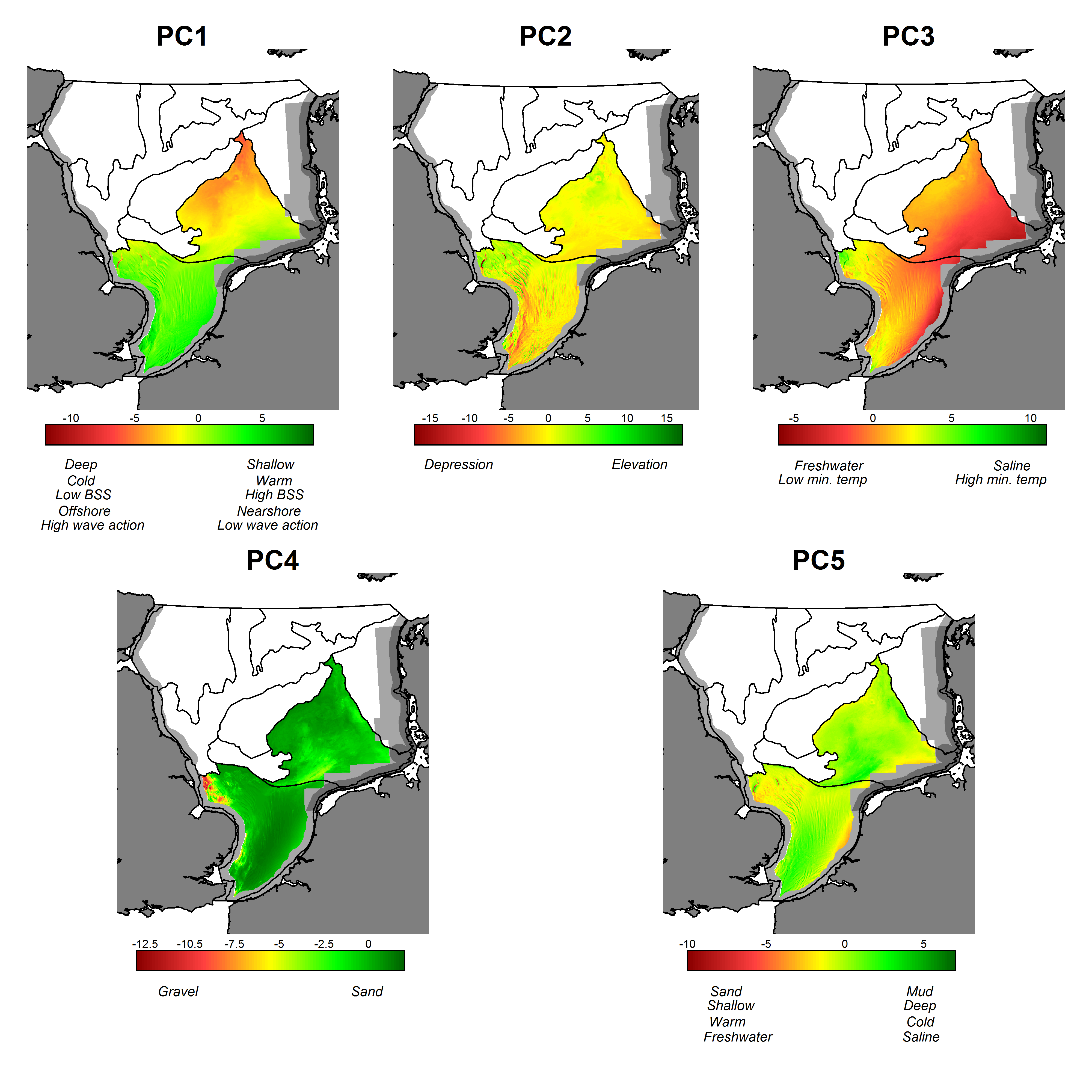

Supplement: S20 Fig — (TIFF) [file pone.0208338.s020.tiff]

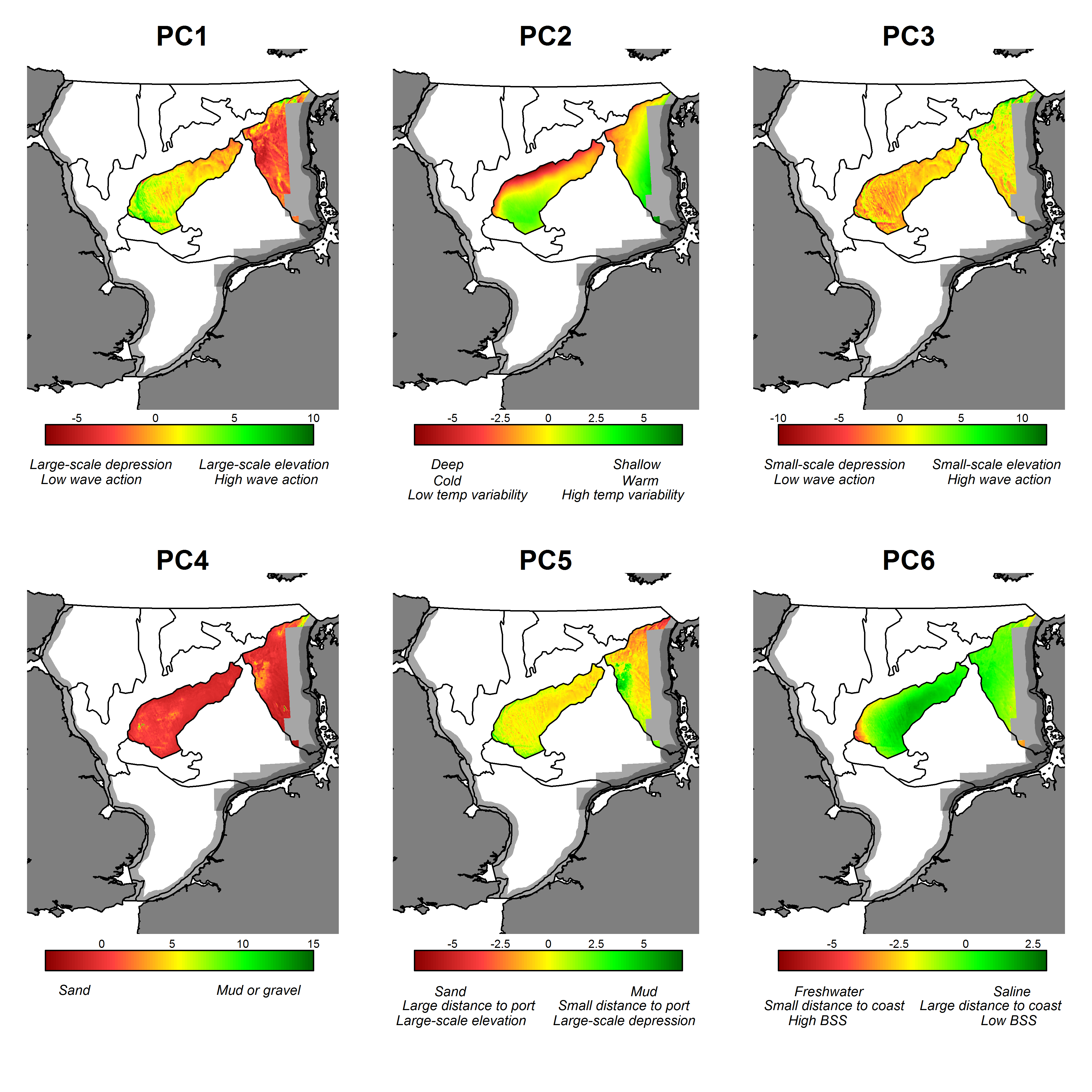

Supplement: S21 Fig — (TIFF) [file pone.0208338.s021.tiff]

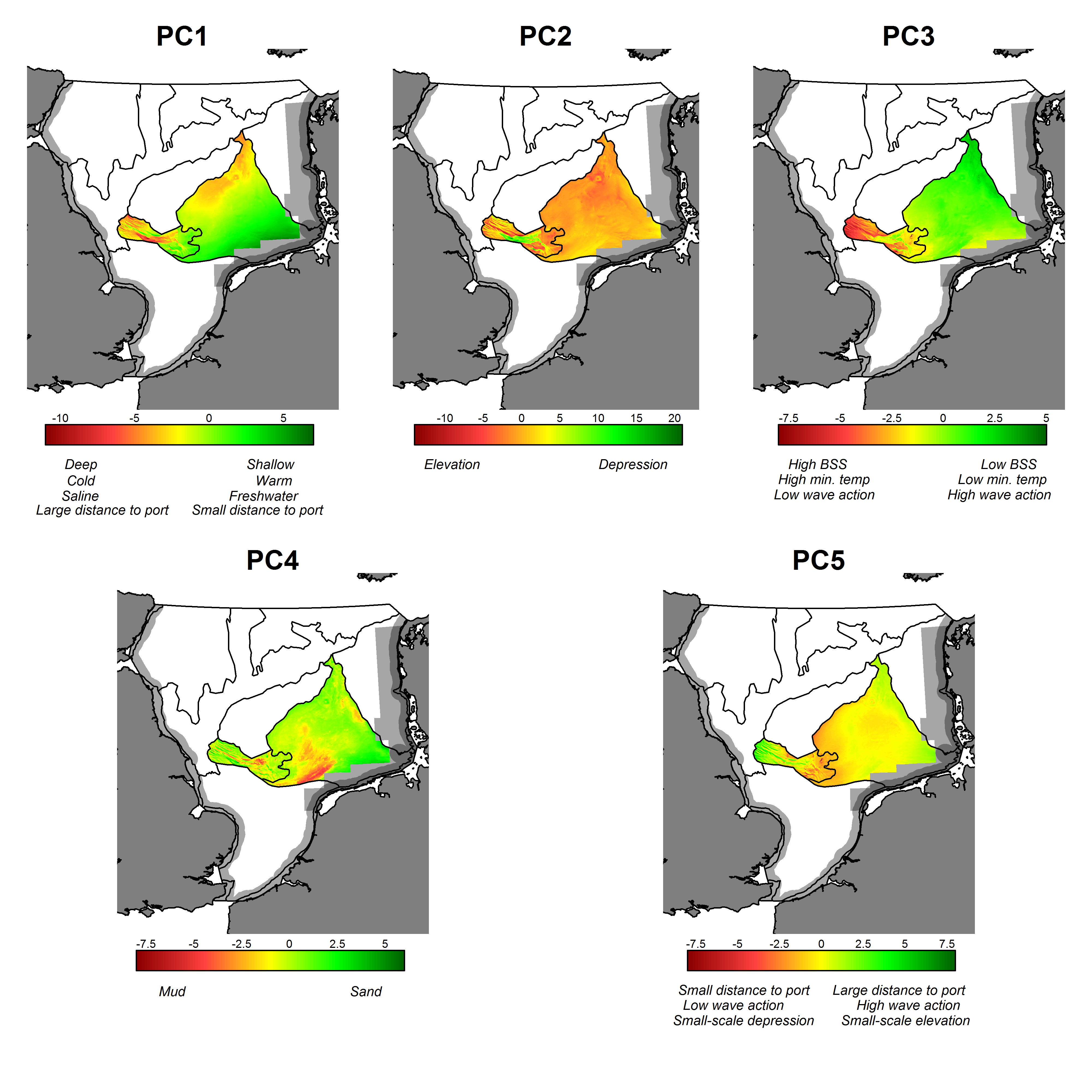

Supplement: S22 Fig — (TIFF) [file pone.0208338.s022.tiff]
