# Supplementary material for: North Sea demersal fisheries prefer specific benthic habitats
Source: PLoS One. 2018 Dec 18;13(12):e0208338. doi: 10.1371/journal.pone.0208338 (PMC6298764; doi:10.1371/journal.pone.0208338)
Supplement: S1 Table — (DOCX) [file pone.0208338.s024.docx]

**S1 Table.** Contribution and permutation importance of all environmental gradients used in the MaxEnt model for Beam-Sole.

| **Environmental gradient** | **Contribution** | **Importance** |
| --- | --- | --- |
| PC 1 | 55.2 | 57.2 |
| PC 2 | 32.7 | 29.8 |
| PC 3 | 1.3 | 4.2 |
| PC 4 | 9.2 | 6.8 |
| PC 5 | 1.6 | 2.0 |
