# Supplementary material for: North Sea demersal fisheries prefer specific benthic habitats
Source: PLoS One. 2018 Dec 18;13(12):e0208338. doi: 10.1371/journal.pone.0208338 (PMC6298764; doi:10.1371/journal.pone.0208338)
Supplement: S2 Table — (DOCX) [file pone.0208338.s025.docx]

**S2 Table.** Contribution and permutation importance of all environmental gradients used in the MaxEnt model for Beam-Plaice.

| **Environmental gradient** | **Contribution** | **Importance** |
| --- | --- | --- |
| PC 1 | 23.8 | 22.2 |
| PC 2 | 25.6 | 20.5 |
| PC 3 | 43.0 | 40.9 |
| PC 4 | 1.0 | 1.7 |
| PC 5 | 5.5 | 1.4 |
| PC 6 | 1.2 | 13.4 |
