# Supplementary material for: North Sea demersal fisheries prefer specific benthic habitats
Source: PLoS One. 2018 Dec 18;13(12):e0208338. doi: 10.1371/journal.pone.0208338 (PMC6298764; doi:10.1371/journal.pone.0208338)
Supplement: S3 Table — (DOCX) [file pone.0208338.s026.docx]

**S3 Table.** Contribution and permutation importance of all environmental gradients used in the MaxEnt model for Otter-Mix.

| **Environmental gradient** | **Contribution** | **Importance** |
| --- | --- | --- |
| PC 1 | 2.3 | 1.7 |
| PC 2 | 47.2 | 40.5 |
| PC 3 | 36.3 | 40.3 |
| PC 4 | 8.3 | 9.3 |
| PC 5 | 5.8 | 8.1 |
