# Supplementary material for: North Sea demersal fisheries prefer specific benthic habitats
Source: PLoS One. 2018 Dec 18;13(12):e0208338. doi: 10.1371/journal.pone.0208338 (PMC6298764; doi:10.1371/journal.pone.0208338)
Supplement: S4 Table — (DOCX) [file pone.0208338.s027.docx]

**S4 Table.** Response curves of the environmental gradients in the MaxEnt model for Beam-Sole, in relation to the abundance of the specific environmental condition.

| **PCA 1** | | | **PCA 2** | | | **PCA 4** | | |
| --- | --- | --- | --- | --- | --- | --- | --- | --- |
| **PCA Value** | **Habitat (%)** | **MaxEnt probability** | **PCA Value** | **Habitat (%)** | **MaxEnt probability** | **PCA Value** | **Habitat (%)** | **MaxEnt probability** |
| -11.25 | 0 | NA | -37.5 | 0 | NA | -12.25 | 0 | NA |
| -10.75 | 0 | NA | -36.5 | 0 | NA | -11.75 | 0.01 | NA |
| -10.25 | 0 | NA | -35.5 | 0 | NA | -11.25 | 0.02 | 0.06 |
| -9.75 | 0 | NA | -34.5 | 0 | NA | -10.75 | 0.05 | 0.06 |
| -9.25 | 0.01 | NA | -33.5 | 0 | NA | -10.25 | 0.07 | 0.06 |
| -8.75 | 0.01 | NA | -32.5 | 0 | NA | -9.75 | 0.08 | 0.06 |
| -8.25 | 0.01 | NA | -31.5 | 0 | NA | -9.25 | 0.1 | 0.06 |
| -7.75 | 0.01 | NA | -30.5 | 0 | NA | -8.75 | 0.11 | 0.06 |
| -7.25 | 0.01 | NA | -29.5 | 0 | NA | -8.25 | 0.11 | 0.06 |
| -6.75 | 0.01 | NA | -28.5 | 0 | NA | -7.75 | 0.11 | 0.06 |
| -6.25 | 0.1 | 0 | -27.5 | 0.01 | NA | -7.25 | 0.14 | 0.06 |
| -5.75 | 0.31 | 0 | -26.5 | 0.01 | NA | -6.75 | 0.17 | 0.06 |
| -5.25 | 0.84 | 0 | -25.5 | 0.01 | NA | -6.25 | 0.21 | 0.06 |
| -4.75 | 1.58 | 0 | -24.5 | 0 | NA | -5.75 | 0.24 | 0.06 |
| -4.25 | 3.57 | 0 | -23.5 | 0 | NA | -5.25 | 0.27 | 0.06 |
| -3.75 | 3.9 | 0.01 | -22.5 | 0 | NA | -4.75 | 0.29 | 0.06 |
| -3.25 | 4.07 | 0.01 | -21.5 | 0 | NA | -4.25 | 0.35 | 0.06 |
| -2.75 | 4.33 | 0.01 | -20.5 | 0 | NA | -3.75 | 0.64 | 0.06 |
| -2.25 | 5.46 | 0.01 | -19.5 | 0.01 | NA | -3.25 | 0.91 | 0.07 |
| -1.75 | 5.64 | 0.01 | -18.5 | 0.01 | NA | -2.75 | 1.01 | 0.07 |
| -1.25 | 5.34 | 0.01 | -17.5 | 0.01 | NA | -2.25 | 1.44 | 0.07 |
| -0.75 | 6.97 | 0.04 | -16.5 | 0.01 | NA | -1.75 | 2.24 | 0.23 |
| -0.25 | 5.39 | 0.15 | -15.5 | 0.01 | 0.97 | -1.25 | 4.4 | 0.23 |
| 0.25 | 5.18 | 0.33 | -14.5 | 0.01 | 0.97 | -0.75 | 8.61 | 0.25 |
| 0.75 | 6.69 | 0.41 | -13.5 | 0.01 | 0.96 | -0.25 | 16.49 | 0.32 |
| 1.25 | 8.08 | 0.44 | -12.5 | 0.01 | 0.94 | 0.25 | 18.01 | 0.38 |
| 1.75 | 8.17 | 0.43 | -11.5 | 0.02 | 0.93 | 0.75 | 28.31 | 0.44 |
| 2.25 | 7.43 | 0.44 | -10.5 | 0.03 | 0.9 | 1.25 | 12.77 | 0.48 |
| 2.75 | 6.1 | 0.46 | -9.5 | 0.04 | 0.88 | 1.75 | 2.85 | 0.61 |
| 3.25 | 5.09 | 0.49 | -8.5 | 0.1 | 0.85 |  |  |  |
| 3.75 | 2.86 | 0.56 | -7.5 | 0.2 | 0.81 |  |  |  |
| 4.25 | 1.41 | 0.7 | -6.5 | 0.33 | 0.77 |  |  |  |
| 4.75 | 0.7 | 0.78 | -5.5 | 0.66 | 0.71 |  |  |  |
| 5.25 | 0.35 | 0.81 | -4.5 | 1.61 | 0.67 |  |  |  |
| 5.75 | 0.16 | 0.83 | -3.5 | 2.76 | 0.65 |  |  |  |
| 6.25 | 0.09 | 0.85 | -2.5 | 4.51 | 0.57 |  |  |  |
| 6.75 | 0.07 | 0.87 | -1.5 | 10.46 | 0.51 |  |  |  |
| 7.25 | 0.04 | 0.89 | -0.5 | 29.04 | 0.41 |  |  |  |
| 7.75 | 0.03 | 0.9 | 0.5 | 27.41 | 0.16 |  |  |  |
| 8.25 | 0 | NA | 1.5 | 11.79 | 0.11 |  |  |  |
| 8.75 | 0 | NA | 2.5 | 5.52 | 0.09 |  |  |  |
|  |  |  | 3.5 | 2.76 | 0.08 |  |  |  |
|  |  |  | 4.5 | 1.21 | 0.07 |  |  |  |
|  |  |  | 5.5 | 0.58 | 0.06 |  |  |  |
|  |  |  | 6.5 | 0.3 | 0.05 |  |  |  |
|  |  |  | 7.5 | 0.2 | 0.04 |  |  |  |
|  |  |  | 8.5 | 0.14 | 0.04 |  |  |  |
|  |  |  | 9.5 | 0.09 | 0.03 |  |  |  |
|  |  |  | 10.5 | 0.06 | 0.03 |  |  |  |
|  |  |  | 11.5 | 0.04 | 0.02 |  |  |  |
|  |  |  | 12.5 | 0.02 | NA |  |  |  |
|  |  |  | 13.5 | 0.01 | NA |  |  |  |
|  |  |  | 14.5 | 0 | NA |  |  |  |
|  |  |  | 15.5 | 0 | NA |  |  |  |
|  |  |  | 16.5 | 0 | NA |  |  |  |
