# Supplementary material for: North Sea demersal fisheries prefer specific benthic habitats
Source: PLoS One. 2018 Dec 18;13(12):e0208338. doi: 10.1371/journal.pone.0208338 (PMC6298764; doi:10.1371/journal.pone.0208338)
Supplement: S5 Table — (DOCX) [file pone.0208338.s028.docx]

**S5 Table.** Response curves of the environmental gradients in the MaxEnt model for Beam-Plaice, in relation to the abundance of the specific environmental condition.

| **PCA 3** | | | **PCA 2** | | | **PCA 1** | | | **PCA 5** | | |
| --- | --- | --- | --- | --- | --- | --- | --- | --- | --- | --- | --- |
| **PCA Value** | **Habitat (%)** | **MaxEnt probability** | **PCA Value** | **Habitat (%)** | **MaxEnt probability** | **PCA Value** | **Habitat (%)** | **MaxEnt probability** | **PCA Value** | **Habitat (%)** | **MaxEnt probability** |
| -9.75 | 0 | NA | -6.7 | 0.02 | NA | -6.75 | 0 | NA | -6.3 | 0 | NA |
| -9.25 | 0 | NA | -6.5 | 0.12 | NA | -6.25 | 0 | NA | -6.1 | 0.01 | NA |
| -8.75 | 0.01 | NA | -6.3 | 0.21 | NA | -5.75 | 0.06 | NA | -5.9 | 0.01 | NA |
| -8.25 | 0.02 | NA | -6.1 | 0.28 | 0.03 | -5.25 | 0.27 | NA | -5.7 | 0.02 | NA |
| -7.75 | 0.03 | NA | -5.9 | 0.28 | 0.04 | -4.75 | 1.29 | 0.03 | -5.5 | 0.02 | NA |
| -7.25 | 0.03 | NA | -5.7 | 0.27 | 0.05 | -4.25 | 1.74 | 0.05 | -5.3 | 0.02 | NA |
| -6.75 | 0.03 | NA | -5.5 | 0.29 | 0.06 | -3.75 | 2.86 | 0.07 | -5.1 | 0.03 | NA |
| -6.25 | 0.05 | NA | -5.3 | 0.35 | 0.08 | -3.25 | 4.76 | 0.1 | -4.9 | 0.04 | NA |
| -5.75 | 0.06 | NA | -5.1 | 0.43 | 0.1 | -2.75 | 6.16 | 0.14 | -4.7 | 0.05 | NA |
| -5.25 | 0.08 | NA | -4.9 | 0.43 | 0.12 | -2.25 | 6.92 | 0.19 | -4.5 | 0.07 | NA |
| -4.75 | 0.18 | 0.02 | -4.7 | 0.46 | 0.14 | -1.75 | 6.49 | 0.23 | -4.3 | 0.13 | NA |
| -4.25 | 0.31 | 0.03 | -4.5 | 0.5 | 0.16 | -1.25 | 5.29 | 0.29 | -4.1 | 0.15 | NA |
| -3.75 | 0.56 | 0.04 | -4.3 | 0.56 | 0.19 | -0.75 | 4.98 | 0.36 | -3.9 | 0.18 | 0.22 |
| -3.25 | 1.05 | 0.06 | -4.1 | 0.65 | 0.22 | -0.25 | 5.71 | 0.4 | -3.7 | 0.17 | 0.23 |
| -2.75 | 2.08 | 0.08 | -3.9 | 0.72 | 0.26 | 0.25 | 6.98 | 0.41 | -3.5 | 0.17 | 0.25 |
| -2.25 | 3.9 | 0.11 | -3.7 | 0.79 | 0.28 | 0.75 | 9.66 | 0.4 | -3.3 | 0.19 | 0.27 |
| -1.75 | 6.68 | 0.15 | -3.5 | 0.86 | 0.3 | 1.25 | 9.49 | 0.39 | -3.1 | 0.19 | 0.29 |
| -1.25 | 10.28 | 0.2 | -3.3 | 0.96 | 0.33 | 1.75 | 8.22 | 0.38 | -2.9 | 0.18 | 0.3 |
| -0.75 | 12.29 | 0.26 | -3.1 | 1.15 | 0.35 | 2.25 | 6.35 | 0.36 | -2.7 | 0.25 | 0.32 |
| -0.25 | 14.19 | 0.33 | -2.9 | 1.28 | 0.38 | 2.75 | 3.99 | 0.34 | -2.5 | 0.35 | 0.33 |
| 0.25 | 14.43 | 0.41 | -2.7 | 1.45 | 0.4 | 3.25 | 2.63 | 0.32 | -2.3 | 0.55 | 0.35 |
| 0.75 | 11.95 | 0.49 | -2.5 | 1.4 | 0.41 | 3.75 | 2.03 | 0.29 | -2.1 | 0.78 | 0.36 |
| 1.25 | 7.84 | 0.57 | -2.3 | 1.49 | 0.42 | 4.25 | 1.48 | 0.26 | -1.9 | 1.02 | 0.37 |
| 1.75 | 4.83 | 0.62 | -2.1 | 1.68 | 0.43 | 4.75 | 1.09 | 0.23 | -1.7 | 1.34 | 0.38 |
| 2.25 | 3.05 | 0.63 | -1.9 | 1.69 | 0.44 | 5.25 | 0.72 | 0.2 | -1.5 | 1.56 | 0.39 |
| 2.75 | 1.94 | 0.65 | -1.7 | 1.89 | 0.45 | 5.75 | 0.39 | 0.17 | -1.3 | 1.95 | 0.4 |
| 3.25 | 1.2 | 0.67 | -1.5 | 2.44 | 0.45 | 6.25 | 0.23 | 0.14 | -1.1 | 2.81 | 0.4 |
| 3.75 | 0.82 | 0.69 | -1.3 | 3.09 | 0.45 | 6.75 | 0.1 | 0.12 | -0.9 | 4.54 | 0.4 |
| 4.25 | 0.59 | 0.7 | -1.1 | 4.19 | 0.44 | 7.25 | 0.05 | NA | -0.7 | 7.98 | 0.41 |
| 4.75 | 0.48 | 0.72 | -0.9 | 4.33 | 0.43 | 7.75 | 0.03 | NA | -0.5 | 10.88 | 0.41 |
| 5.25 | 0.3 | 0.73 | -0.7 | 3.94 | 0.42 | 8.25 | 0.02 | NA | -0.3 | 11.42 | 0.41 |
| 5.75 | 0.17 | 0.75 | -0.5 | 4.53 | 0.4 | 8.75 | 0.01 | NA | -0.1 | 10.32 | 0.4 |
| 6.25 | 0.14 | 0.76 | -0.3 | 4.14 | 0.39 | 9.25 | 0 | NA | 0.1 | 8.48 | 0.4 |
| 6.75 | 0.11 | 0.78 | -0.1 | 2.83 | 0.37 |  |  |  | 0.3 | 6.81 | 0.39 |
| 7.25 | 0.09 | 0.79 | 0.1 | 2.99 | 0.35 |  |  |  | 0.5 | 5.27 | 0.39 |
| 7.75 | 0.06 | 0.8 | 0.3 | 3.05 | 0.32 |  |  |  | 0.7 | 4.13 | 0.38 |
| 8.25 | 0.05 | 0.82 | 0.5 | 3.36 | 0.3 |  |  |  | 0.9 | 3.27 | 0.37 |
| 8.75 | 0.04 | 0.83 | 0.7 | 2.92 | 0.27 |  |  |  | 1.1 | 2.63 | 0.36 |
| 9.25 | 0.03 | 0.84 | 0.9 | 2.75 | 0.24 |  |  |  | 1.3 | 2.16 | 0.35 |
| 9.75 | 0.02 | NA | 1.1 | 2.85 | 0.21 |  |  |  | 1.5 | 1.71 | 0.33 |
| 10.25 | 0.01 | NA | 1.3 | 3.01 | 0.19 |  |  |  | 1.7 | 1.53 | 0.32 |
| 10.75 | 0.01 | NA | 1.5 | 3.21 | 0.16 |  |  |  | 1.9 | 1.08 | 0.3 |
| 11.25 | 0 | NA | 1.7 | 3.44 | 0.13 |  |  |  | 2.1 | 0.73 | 0.28 |
| 11.75 | 0 | NA | 1.9 | 3.58 | 0.11 |  |  |  | 2.3 | 0.59 | 0.27 |
|  |  |  | 2.1 | 3.36 | 0.09 |  |  |  | 2.5 | 0.49 | 0.25 |
|  |  |  | 2.3 | 3.12 | 0.07 |  |  |  | 2.7 | 0.4 | 0.23 |
|  |  |  | 2.5 | 2.56 | 0.06 |  |  |  | 2.9 | 0.32 | 0.21 |
|  |  |  | 2.7 | 2.33 | 0.05 |  |  |  | 3.1 | 0.32 | 0.19 |
|  |  |  | 2.9 | 1.7 | 0.03 |  |  |  | 3.3 | 0.32 | 0.18 |
|  |  |  | 3.1 | 1.04 | 0.03 |  |  |  | 3.5 | 0.31 | 0.16 |
|  |  |  | 3.3 | 1.02 | 0.02 |  |  |  | 3.7 | 0.33 | 0.14 |
|  |  |  | 3.5 | 0.8 | 0.01 |  |  |  | 3.9 | 0.35 | 0.13 |
|  |  |  | 3.7 | 0.78 | 0.01 |  |  |  | 4.1 | 0.31 | 0.11 |
|  |  |  | 3.9 | 0.66 | 0.01 |  |  |  | 4.3 | 0.28 | 0.1 |
|  |  |  | 4.1 | 0.55 | 0.01 |  |  |  | 4.5 | 0.23 | 0.08 |
|  |  |  | 4.3 | 0.32 | 0 |  |  |  | 4.7 | 0.18 | NA |
|  |  |  | 4.5 | 0.2 | 0 |  |  |  | 4.9 | 0.12 | NA |
|  |  |  | 4.7 | 0.11 | 0 |  |  |  | 5.1 | 0.1 | NA |
|  |  |  | 4.9 | 0 | NA |  |  |  | 5.3 | 0.07 | NA |
|  |  |  | 5.1 | 0 | NA |  |  |  | 5.5 | 0.05 | NA |
|  |  |  | 5.3 | 0.03 | NA |  |  |  | 5.7 | 0.02 | NA |
|  |  |  | 5.5 | 0.09 | NA |  |  |  | 5.9 | 0.01 | NA |
|  |  |  | 5.7 | 0.08 | NA |  |  |  | 6.1 | 0 | NA |
|  |  |  | 5.9 | 0.1 | NA |  |  |  | 6.3 | 0 | NA |
|  |  |  | 6.1 | 0.1 | NA |  |  |  | 6.5 | 0 | NA |
|  |  |  | 6.3 | 0.12 | NA |  |  |  |  |  |  |
|  |  |  | 6.5 | 0.05 | NA |  |  |  |  |  |  |
|  |  |  | 6.7 | 0 | NA |  |  |  |  |  |  |
