# Supplementary material for: North Sea demersal fisheries prefer specific benthic habitats
Source: PLoS One. 2018 Dec 18;13(12):e0208338. doi: 10.1371/journal.pone.0208338 (PMC6298764; doi:10.1371/journal.pone.0208338)
Supplement: S6 Table — (DOCX) [file pone.0208338.s029.docx]

**S6 Table.** Response curves of the environmental gradients in the MaxEnt model for Otter-Mix, in relation to the abundance of the specific environmental condition.

| **PCA 2** | | | **PCA 3** | | | **PCA 4** | | | **PCA 5** | | |
| --- | --- | --- | --- | --- | --- | --- | --- | --- | --- | --- | --- |
| **PCA Value** | **Habitat (%)** | **MaxEnt probability** | **PCA Value** | **Habitat (%)** | **MaxEnt probability** | **PCA Value** | **Habitat (%)** | **MaxEnt probability** | **PCA Value** | **Habitat (%)** | **MaxEnt probability** |
| -13.75 | 0 | NA | -23.75 | 0 | NA | -7.3 | 0 | NA | -31.5 | 0 | NA |
| -13.25 | 0 | NA | -23.25 | 0 | NA | -7.1 | 0 | NA | -30.5 | 0 | NA |
| -12.75 | 0.01 | NA | -22.75 | 0 | NA | -6.9 | 0 | NA | -29.5 | 0 | NA |
| -12.25 | 0.01 | NA | -22.25 | 0 | NA | -6.7 | 0 | NA | -28.5 | 0 | NA |
| -11.75 | 0.01 | NA | -21.75 | 0 | NA | -6.5 | 0 | NA | -27.5 | 0 | NA |
| -11.25 | 0.02 | NA | -21.25 | 0 | NA | -6.3 | 0 | NA | -26.5 | 0 | NA |
| -10.75 | 0.02 | NA | -20.75 | 0 | NA | -6.1 | 0 | NA | -25.5 | 0 | NA |
| -10.25 | 0.02 | NA | -20.25 | 0 | NA | -5.9 | 0 | NA | -24.5 | 0 | NA |
| -9.75 | 0.03 | NA | -19.75 | 0 | NA | -5.7 | 0 | NA | -23.5 | 0 | NA |
| -9.25 | 0.03 | NA | -19.25 | 0 | NA | -5.5 | 0 | NA | -22.5 | 0 | NA |
| -8.75 | 0.04 | NA | -18.75 | 0 | NA | -5.3 | 0.01 | NA | -21.5 | 0 | NA |
| -8.25 | 0.05 | NA | -18.25 | 0 | NA | -5.1 | 0.02 | NA | -20.5 | 0 | NA |
| -7.75 | 0.06 | 0.01 | -17.75 | 0 | NA | -4.9 | 0.07 | 0.36 | -19.5 | 0 | NA |
| -7.25 | 0.07 | 0.01 | -17.25 | 0 | NA | -4.7 | 0.16 | 0.35 | -18.5 | 0 | NA |
| -6.75 | 0.08 | 0.01 | -16.75 | 0 | NA | -4.5 | 0.24 | 0.34 | -17.5 | 0 | NA |
| -6.25 | 0.1 | 0.02 | -16.25 | 0 | NA | -4.3 | 0.3 | 0.33 | -16.5 | 0 | NA |
| -5.75 | 0.12 | 0.02 | -15.75 | 0 | NA | -4.1 | 0.34 | 0.33 | -15.5 | 0 | NA |
| -5.25 | 0.15 | 0.02 | -15.25 | 0 | NA | -3.9 | 0.39 | 0.32 | -14.5 | 0 | NA |
| -4.75 | 0.26 | 0.03 | -14.75 | 0 | NA | -3.7 | 0.36 | 0.31 | -13.5 | 0 | NA |
| -4.25 | 0.47 | 0.03 | -14.25 | 0 | NA | -3.5 | 0.36 | 0.31 | -12.5 | 0 | NA |
| -3.75 | 0.76 | 0.04 | -13.75 | 0 | NA | -3.3 | 0.39 | 0.3 | -11.5 | 0 | NA |
| -3.25 | 1.19 | 0.04 | -13.25 | 0 | NA | -3.1 | 0.42 | 0.29 | -10.5 | 0 | NA |
| -2.75 | 2.03 | 0.05 | -12.75 | 0 | NA | -2.9 | 0.42 | 0.29 | -9.5 | 0 | NA |
| -2.25 | 4.31 | 0.06 | -12.25 | 0 | NA | -2.7 | 0.58 | 0.28 | -8.5 | 0 | NA |
| -1.75 | 6.54 | 0.07 | -11.75 | 0 | NA | -2.5 | 0.82 | 0.27 | -7.5 | 0 | NA |
| -1.25 | 10.72 | 0.12 | -11.25 | 0 | NA | -2.3 | 1.06 | 0.26 | -6.5 | 0.01 | NA |
| -0.75 | 14.65 | 0.16 | -10.75 | 0 | NA | -2.1 | 1.16 | 0.25 | -5.5 | 0.02 | 0.68 |
| -0.25 | 13.41 | 0.18 | -10.25 | 0 | NA | -1.9 | 1.47 | 0.24 | -4.5 | 0.06 | 0.59 |
| 0.25 | 12.55 | 0.2 | -9.75 | 0 | NA | -1.7 | 2.04 | 0.24 | -3.5 | 0.21 | 0.5 |
| 0.75 | 11.44 | 0.25 | -9.25 | 0 | NA | -1.5 | 2.36 | 0.23 | -2.5 | 1.49 | 0.41 |
| 1.25 | 7.86 | 0.31 | -8.75 | 0 | NA | -1.3 | 2.27 | 0.23 | -1.5 | 13.48 | 0.31 |
| 1.75 | 3.6 | 0.34 | -8.25 | 0 | NA | -1.1 | 2.65 | 0.22 | -0.5 | 34.32 | 0.2 |
| 2.25 | 2.74 | 0.37 | -7.75 | 0 | NA | -0.9 | 2.94 | 0.21 | 0.5 | 38.17 | 0.23 |
| 2.75 | 2 | 0.4 | -7.25 | 0.01 | NA | -0.7 | 3.21 | 0.21 | 1.5 | 9.15 | 0.15 |
| 3.25 | 0.74 | 0.43 | -6.75 | 0.06 | NA | -0.5 | 4.57 | 0.2 | 2.5 | 1.67 | 0.09 |
| 3.75 | 0.47 | 0.46 | -6.25 | 0.24 | 0.04 | -0.3 | 7.14 | 0.2 | 3.5 | 0.96 | 0.07 |
| 4.25 | 0.39 | 0.49 | -5.75 | 0.51 | 0.04 | -0.1 | 8.38 | 0.19 | 4.5 | 0.35 | 0.05 |
| 4.75 | 0.32 | 0.52 | -5.25 | 0.82 | 0.04 | 0.1 | 9.45 | 0.19 | 5.5 | 0.08 | NA |
| 5.25 | 0.25 | 0.55 | -4.75 | 1.28 | 0.05 | 0.3 | 9.31 | 0.18 | 6.5 | 0.01 | NA |
| 5.75 | 0.22 | 0.57 | -4.25 | 1.84 | 0.05 | 0.5 | 8.89 | 0.18 | 7.5 | 0 | NA |
| 6.25 | 0.2 | 0.58 | -3.75 | 1.41 | 0.06 | 0.7 | 7.29 | 0.17 |  |  |  |
| 6.75 | 0.21 | 0.59 | -3.25 | 1.55 | 0.07 | 0.9 | 4.6 | 0.17 |  |  |  |
| 7.25 | 0.24 | 0.6 | -2.75 | 1.37 | 0.07 | 1.1 | 3.44 | 0.13 |  |  |  |
| 7.75 | 0.28 | 0.61 | -2.25 | 1.33 | 0.08 | 1.3 | 2.32 | 0.09 |  |  |  |
| 8.25 | 0.24 | 0.61 | -1.75 | 3.35 | 0.1 | 1.5 | 1.89 | 0.09 |  |  |  |
| 8.75 | 0.19 | 0.62 | -1.25 | 5.38 | 0.11 | 1.7 | 1.63 | 0.08 |  |  |  |
| 9.25 | 0.15 | 0.63 | -0.75 | 9.31 | 0.13 | 1.9 | 1.24 | 0.08 |  |  |  |
| 9.75 | 0.13 | 0.63 | -0.25 | 11.01 | 0.15 | 2.1 | 1.16 | 0.07 |  |  |  |
| 10.25 | 0.11 | 0.64 | 0.25 | 12.88 | 0.17 | 2.3 | 1.19 | 0.07 |  |  |  |
| 10.75 | 0.09 | 0.64 | 0.75 | 24.24 | 0.2 | 2.5 | 0.96 | 0.06 |  |  |  |
| 11.25 | 0.07 | 0.65 | 1.25 | 11 | 0.24 | 2.7 | 0.77 | 0.05 |  |  |  |
| 11.75 | 0.07 | 0.65 | 1.75 | 3.52 | 0.34 | 2.9 | 0.56 | 0.05 |  |  |  |
| 12.25 | 0.06 | 0.65 | 2.25 | 5.56 | 0.48 | 3.1 | 0.41 | 0.04 |  |  |  |
| 12.75 | 0.05 | 0.65 | 2.75 | 3.03 | 0.56 | 3.3 | 0.35 | 0.04 |  |  |  |
| 13.25 | 0.04 | 0.65 | 3.25 | 0.25 | 0.57 | 3.5 | 0.21 | 0.03 |  |  |  |
| 13.75 | 0.03 | 0.65 | 3.75 | 0.02 | NA | 3.7 | 0.11 | 0.03 |  |  |  |
| 14.25 | 0.03 | 0.65 | 4.25 | 0 | NA | 3.9 | 0.04 | NA |  |  |  |
| 14.75 | 0.02 | 0.65 |  |  |  | 4.1 | 0.02 | NA |  |  |  |
| 15.25 | 0.02 | 0.65 |  |  |  | 4.3 | 0.01 | NA |  |  |  |
| 15.75 | 0.01 | 0.64 |  |  |  | 4.5 | 0.01 | NA |  |  |  |
| 16.25 | 0.01 | 0.64 |  |  |  | 4.7 | 0 | NA |  |  |  |
| 16.75 | 0.01 | 0.64 |  |  |  | 4.9 | 0 | NA |  |  |  |
| 17.25 | 0.01 | NA |  |  |  | 5.1 | 0 | NA |  |  |  |
| 17.75 | 0.01 | NA |  |  |  | 5.3 | 0 | NA |  |  |  |
| 18.25 | 0.01 | NA |  |  |  | 5.5 | 0 | NA |  |  |  |
| 18.75 | 0 | NA |  |  |  |  |  |  |  |  |  |
| 19.25 | 0 | NA |  |  |  |  |  |  |  |  |  |
| 19.75 | 0 | NA |  |  |  |  |  |  |  |  |  |
| 20.25 | 0 | NA |  |  |  |  |  |  |  |  |  |
| 20.75 | 0 | NA |  |  |  |  |  |  |  |  |  |
